# Niche-DE: niche-differential gene expression analysis in spatial transcriptomics data identifies context-dependent cell-cell interactions

**DOI:** 10.1186/s13059-023-03159-6

**Published:** 2024-01-12

**Authors:** Kaishu Mason, Anuja Sathe, Paul R. Hess, Jiazhen Rong, Chi-Yun Wu, Emma Furth, Katalin Susztak, Jonathan Levinsohn, Hanlee P. Ji, Nancy Zhang

**Affiliations:** 1 Department of Statistics and Data Science, The Wharton School, University of Pennsylvania, Philadelphia, USA; 2 Division of Oncology, Department of Medicine, Stanford University School of Medicine, Stanford, CA USA; 3 Genomics and Computational Biology Graduate Program, Perelman School of Medicine, University of Pennsylvania, Philadelphia, USA; 4 The Gladstone Institute, San Francisco, USA; 5 Department of Pathology and Laboratory Medicine, Perelman School of Medicine, University of Pennsylvania, Philadelphia, USA; 6 Department of Medicine, University of Pennsylvania Perelman School of Medicine, Philadelphia, PA USA

## Abstract

Existing methods for analysis of spatial transcriptomic data focus on delineating the global gene expression variations of cell types across the tissue, rather than local gene expression changes driven by cell-cell interactions. We propose a new statistical procedure called niche-differential expression (niche-DE) analysis that identifies cell-type-specific niche-associated genes, which are differentially expressed within a specific cell type in the context of specific spatial niches. We further develop niche-LR, a method to reveal ligand-receptor signaling mechanisms that underlie niche-differential gene expression patterns. Niche-DE and niche-LR are applicable to low-resolution spot-based spatial transcriptomics data and data that is single-cell or subcellular in resolution.

**Supplementary Information:**

The online version contains supplementary material available at 10.1186/s13059-023-03159-6.

## Background

Cells within a tissue must constantly work together to adapt to changing environments. In order to coordinate these changes, communication mechanisms such as paracrine signaling are used. In paracrine signaling, a cell releases chemical substances such as hormones, neurotransmitters, and growth factors that bind to a specific protein receptor on neighboring cells, eliciting a change in function of those cells. As such, specific gene expression programs in a cell may be driven by the activities of other cells in its niche.

Spatial transcriptomic (ST) technologies are rapidly maturing, enabling the high resolution in situ measurement of gene expression at the transcriptome scale. Such technologies include Merfish [1], SeqFISH+ [2], Visium [3], Slide-seq [4], and Stereo-seq [5]. To mine this data, computational methods have been proposed for image segmentation [6], technical artifact removal [7], spot deconvolution for spot-based data [8, 9], spatially variable gene detection [10–13], neighborhood detection [14–18], cell-cell interaction analysis [19–23], and other analyses [6, 24–26]. In particular, the detection of spatially variable genes, defined as genes displaying clear spatial patterns in expression, has become a standard analysis step. Spatially variable genes can be used to aid in tasks routinely performed by histopathology, such as the visualization of tissue architecture, and further to identify cell types that have distinct spatial localization. Once the distribution of cell types have been determined at a macro level, it is often of interest to interrogate local, cell-type-specific interactions [27]. By design, spatially variable gene analysis is geared towards the identification of global patterns in gene expression, not local interactions between cell types. For example, in cancer tissue, spatially variable genes allow us to demarcate cancer vs normal tissue regions; however, it is yet unclear how to identify and assess the significance of local patterns such as niche signaling between tumor and immune cells.

Local interactions between cells, such as those based on signaling pathways or chemical and biomechanical remodeling of the extracellular matrix, are highly cell type specific. Thus, we explore cell-type-specific models for the dependence of a cell’s gene expression program on its local microenvironment niche. In our analyses, we define a cell’s niche as the cell-type composition of its local neighborhood, where the range of the local neighborhood is determined by a kernel function. We expect that a cell’s niche affects its gene expression profile, and to this end, propose a new statistical procedure called niche-differential expression (niche-DE) analysis. Niche-DE identifies cell-type-specific niche-associated genes, defined as genes whose expression within specific cell type(s) is significantly up- or downregulated in the context of specific spatial niches, as compared to their cell-type-specific mean expression. Although niche-DE is conceptually defined at the single-cell level, we derive an equivalent model for the recovery of niche-DE genes from lower-resolution spatial transcriptomic data where each observation is a spot (or region of interest) containing a mixture of cell types. To ensure rigorous and reproducible analyses, we propose an interpretable framework for FDR control that reports significant signals at three levels (gene, cell type, and interaction level). Through simulations, we show that the method is robust to overdispersion and spot swapping.

We would like to highlight the differences between niche-differential expression analysis and common spatial analyses such as spatially variable gene analysis and cell-cell interaction analysis. Spatially variable gene analysis finds genes that show expression patterns that correlate with some spatial pattern [10–13], while cell-cell interaction analysis looks for cell types that colocalize with each other more than expected by random chance [19–23]. While the former does not explicitly consider the colocalization of cell types present in the tissue, the latter is a function of the cell types and their locations with no regard to gene expression. In contrast, niche-differential expression analysis finds genes in an index cell type (e.g., macrophages) that show expression patterns that correlate with its colocalization with another cell type (e.g., tumor cells). This gives insight to how colocalization of two cell types affects their gene expression state. The method that is most related to niche-DE in terms of analysis goal is C-Side [28], but that also has substantial differences, which we will discuss in the “Results” and “Discussion”.

One attractive feature of niche-differential expression analysis versus spatially variable gene analysis is that the former is easier to interpret across multiple tissue slides. A statement such as “gene $X$ is spatially variable, enriched in the left part of the slide” is difficult to generalize across samples due to differences in frames of reference. In contrast, a statement such as “macrophages upregulate expression of a given gene $X$ when situated near tumor cells” can be applied and tested across multiple tissue slices. As such, niche-DE analyses can naturally integrate data from multiple tissue slides and across patients, with strategies to correct for tissue- and slide-batch effects.

When niche-differentially expressed genes are identified, a natural follow-up inquiry is whether a known cell-cell signaling mechanism is responsible for their up- or downregulation. Towards this goal, we developed niche-LR, a procedure that integrates niche-DE statistics with Niche-net ligand-target matrices [27] to identify ligand-receptor signaling channels between cell types. We also derive rigorous test statistics for this step and show that niche-LR can recover cell-cell signaling interactions for spot-based spatial transcriptomic data where each spot may be a mixture of cells.

We illustrate and benchmark niche-DE on simulated CosMx SMI [29], Slide-seq [4], and Xenium [30]. In an extensive analysis of 10× Visium data consisting of liver metastases of colorectal cancer from 5 patients, Niche-DE identifies known and novel marker genes for tumor-associated fibroblasts and macrophages and elucidates ligand-receptor crosstalk patterns between tumor cells, macrophages and fibroblasts. These findings are corroborated by parallel analyses of single-cell RNA sequencing (scRNA-seq) and CODEX data for the same tissue type, and in one case, from the same patient. As an example of niche-DE application in a non-cancer setting, we also analyzed an example data set from chronic kidney disease, where niche-DE uncovered well-known disease-associated gene markers for fibroblast transformation and proximal tubule cell injury.

## Results

### Single-cell niche-differential gene expression model

We start by describing the niche-DE model, first for data of single-cell resolution and then for data of lower resolution. We then summarize the framework for FDR control and bandwidth selection. Details of the model and algorithm are given in the “Methods” section.

**Fig. 1:**
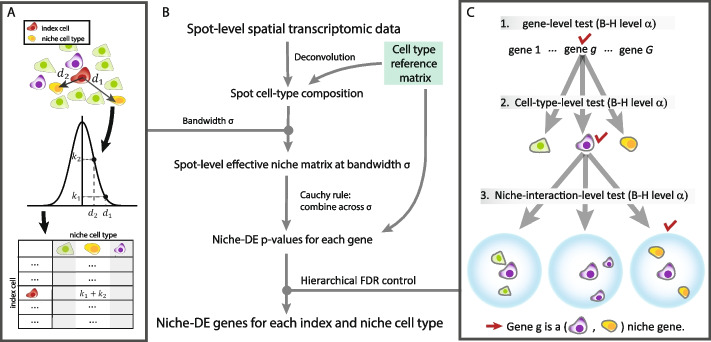
**A** Schematic of effective niche calculation: We aim to quantify the cell-type composition of each cell’s neighborhood. For each index cell, we calculate the pairwise kernel distance similarity between itself and each other cell in the sample. We use a Gaussian kernel with bandwidth $\sigma$. The effective niche for the index cell is a vector with dimension equal to the number of unique cell types in the sample where index $i$ represents the sum of kernel similarities between the index cell and the cells of type $i$. **B** Schematic of niche-DE pipeline: To perform niche-DE, we first perform deconvolution/cell-type identification of our data. We then calculate the effective niche using a Gaussian kernel of bandwidth $\sigma$. We then apply the regression-based niche-DE model using the effective niche calculated in the previous step. If one desires, they can repeat this step for multiple bandwidths. Using the Cauchy combination test across the different kernels used, we can calculate a *p*-value for testing whether gene $g$ is an $(i,n)$ niche gene for all genes $g$ and index-niche cell-type pairs. **C** FDR control: To guarantee correct FDR control, we utilize the hierarchical Benjamini-Hochberg procedure [33]. We first test if a gene shows evidence of being a niche-DE gene in any index-niche pair. This results in a *p*-value for each gene. We then apply the BH procedure to this set of genes. For a gene whose adjusted *p*-value is below the cutoff value, we test if it a niche gene in with index cell type $i$. Testing across all $T$ unique cell types in our sample, we get $T$ cell-type-specific *p*-values for each gene $g$ that is tested at this level. We then apply the Benjamini-Hochberg correction at level $\alpha$ across these *p*-values for each gene. For all gene, index cell-type pairs $(g,i)$ that are significant after correction, we proceed to test if gene $g$ is an $(i,n)$ niche gene for each niche cell type $n$. After applying the Benjamini-Hochberg correction at level $\alpha$ across all $T$*p*-values for each $(g,i)$ pair, if a $\left(g,i,n\right)$ set has a *p*-value below the cutoff value, we conclude that gene $g$ is an $(i,n)$ niche gene

For spatial transcriptomic data at *single-cell* resolution, we assume that the cells have already been labeled by type, e.g., through the use of marker genes or a reference-based label transfer method [31, 32]. Let ${Y}_{c,g}$ be the observed count for gene $g\in \{1,\dots ,G\}$ in cell $c\in \{1,\dots ,C\}$, ${T}_{c}\in \{1,\dots ,T\}$ be the type of cell $c$, and ${\mu }_{t,g}=E({Y}_{c,g}|{T}_{c}=t)$ be the expected expression of gene $g$ in a cell of type $t$. For each cell, we summarize its spatial neighborhood through the computation of a kernel-smoothed density of cell-type compositions centered at the cell (Fig. 1A):

$${N}_{\sigma ,c,t}= \sum_{{c}{\prime}=1}^{C}{K}_{\sigma }\left({d}_{c,{c}{\prime}}\right)I({t}_{{c}{\prime}}=t),$$

where ${d}_{c,{c}^\prime}$ is the physical distance between cells $c, {c}^\prime$ and ${K}_{\sigma }\left(d\right)$ is a kernel of bandwidth $\sigma$, e.g., ${K}_{\sigma }\left(d\right)=\frac{1}{\sigma }\phi (\frac{d}{\sigma })$, where $\phi$ is the Gaussian filter. We call ${N}_{\sigma ,c}=({N}_{\sigma ,c,1}, \dots , {N}_{\sigma ,c,T})$ the effective niche cell-type composition of cell $c$ with kernel bandwidth $\sigma$. In defining ${N}_{\sigma ,c}$, we refer to cell $c$ as the index cell (Fig. 1A). In summary, we characterize the spatial niche of a cell as a $T$ dimensional vector where index $j$ measures how much of cell type $j$ is in the neighborhood of the index cell. For example, a cell that is surrounded by tumor cells will have an effective niche that is 0 in all indices except for the one that corresponds to tumor cells. Meanwhile, a cell with a neighborhood dense in different immune cell types will have an effective niche with many non-zero entries. The exact density of each cell type within the neighborhood will determine the magnitude of the entry in the effective niche. We consider first the case where the bandwidth $\sigma$ is given, and later discuss the pooling of evidence across multiple $\sigma$.

For modeling sequencing-based single-cell data, the negative binomial model has been well studied and shown to be a useful model [34, 35]. Thus, in the single-cell niche-differential gene expression model, the expression of gene $g$ in index cell $c$ is negative binomial-distributed with dispersion ${\gamma }_{g}$ and mean that varies according to both the index cell type and the effective niche:

1 $${\text{log}}E\left[{Y}_{c,g}|{T}_{c},{N}_{\sigma ,c}\right]={\text{log}}{\mu }_{{T}_{c},g}+\sum\limits_{n=1}^{T}{N}_{\sigma ,c,n}{\beta }_{{\sigma ,t}_{c},n}^{g}$$

where ${\mu }_{{T}_{c},g}=E[{Y}_{c,g}|{T}_{c}]$ is the global mean of gene $g$ in cells of type ${T}_{c}$. Let $i\in \{1,\dots ,T\}$ be the index cell type, and $n\in \{1,\dots , T\}$ be the niche cell type, our goal is to estimate the cell-type-specific niche-differential expression parameters $\{{\beta }_{\sigma ,i,n}^{g}\}$, and to identify those genes $g$ and index-niche cell-type combinations $(i,n)$ where ${\beta }_{\sigma ,i,n}^{g}\ne 0$. A significant test against the null hypothesis ${\beta }_{\sigma ,i,n}^{g}=0$ means that, when the index cell is of type $i$, the enrichment of cell type $n$ in the effective niche is associated with a significant change in the expression of gene $g$ within the index cell. In this case, we call gene $g$ an ${\left(i,n\right)}^{+}$ niche gene if the association is positive and an ${\left(i,n\right)}^{-}$ niche gene if the association is negative. Note that the relationship between niche and index cell type is asymmetric.

### Detecting niche-differential expression from spot-level data

Now consider the case where, instead of single cells, we observe spots $s\in \{1,\dots ,S\}$, where each spot may be a mixture of cells of different types. Let ${X}_{s,g}$ be the count of gene $g$ in spot $s$. In “Methods,” we show that model (1) is approximately equivalent to the following spot-level model:

2 $${\text{log}}\left({X}_{s,g}\right)={\text{log}}\left({\mu }_{s,g}\right)+\sum\limits_{\left\{i\right\}}\sum\limits_{\left\{n\right\}}{p}_{s,i,g}{N}_{\sigma ,s,n}{\beta }_{\sigma ,i,n}^{g}$$

where ${\mu }_{s,g}= {\sum }_{c:s\left(c\right)=s}{\mu }_{{T}_{c},g}$ is the expected expression of gene $g$ in spot $s$ given the true cell-type composition of spot $s$, ${p}_{s,i,g}={\left({\mu }_{s,g}\right)}^{-1}\sum_{c:s\left(c\right)=s}{\mu }_{{t}_{c},g}I({t}_{c}=i)$ is the expected proportion of gene $g$’s expression in spot $s$ that originate from cell type $i$, and ${N}_{\sigma ,s,n}$ is the concentration of cell type $n$ in the effective niche of spot $s$. As shown in Fig. 1B, we start by computing the terms ${\mu }_{s,g}, {p}_{s,i,g}$ and ${N}_{\sigma ,s,n}$ via spot deconvolution [8]. Then, the terms ${\beta }_{\sigma ,i,n}^{g}$ can be estimated along with their corresponding *p*-values ${p}_{\sigma ,i,n}^{g}$ via negative binomial regression. It is important to note that, despite the fact that model (2) is fit using lower-resolution spot-level data, the set-up of the model ensures that the interpretation of the parameters ${\beta }_{\sigma ,t,{t}^\prime}^{g}$ are exactly the same as for model (1). We stress that in the case of spot-level data, a good deconvolution must be performed. This is because errors in the deconvolution will lead to an incorrect effective niche calculation which is the primary covariate in the niche-DE model. Therefore, we assume that a satisfactory deconvolution has been performed prior to performing niche-DE.

Among published methods, the method that is most similar to niche-DE in terms of analysis goal is C-Side [28]. There are important methodological differences between niche-DE and C-side. By starting from a single-cell model and then using it to derive a spot-level model, niche-DE gives explicit interpretation of the parameter ${\beta }_{i,n}^{g}$, which is the increase in expression of gene g in index cell type $i$ per unit increase in the niche proportion of cell type $n$. Although C-Side also aims to detect cell-type-specific differentially expressed genes, its model does not admit to this explicit interpretation of parameters. Niche-DE also accounts for the average library size of each cell type in performing differential expression analysis. This is important because C-side assumes that the change in gene expression for a spot is equal to the average change across all cell types in the spot. Niche-DE also differs from C-side in the way that it treats multiple cell types, the unknown spatial neighborhood size, and the massively parallel multiple tests, as we detail in the “Discussion”.

### Hierarchical false discovery rate control

For each gene, hypothesis tests are performed for ${T}^{2}$ parameters: one parameter ${\beta }_{\sigma ,i,n}^{g}$ for each index-niche cell-type pair. Across $G$ genes, this amounts to a parallel screen of $G{T}^{2}$*p*-values. As shown in Fig. 1C, we control the false discovery rate (FDR) through a hierarchical Benjamini-Hochberg procedure [33]. Specifically, we start with a gene-level test, to identify genes that are niche-DE in *at least one* of the ${T}^{2}$ possible cell-type configurations. This is achieved through applying Browns method [36] on the set of *p*-values obtained through testing the null hypothesis ${\beta }_{\sigma ,i,n}^{g}=0$ for all $i,n$. Thus for each $\sigma$, we have a gene specific *p*-value ${p}_{g}^{\sigma }$. We combine these *p*-values into a single *p*-value, ${p}_{g}$, via the Cauchy combination procedure [37]. A Benjamini-Hochberg correction at level $\alpha$ is applied to the gene-level *p*-values. For genes that are rejected at this level, we proceed to test at the cell type level: for each cell type $i,$ we again apply Brown’s method on the set of *p*-values obtained through testing the null hypothesis ${\beta }_{\sigma ,i,n}^{g}=0$ for all niche cell types $n$.Thus, for each $\sigma ,$ we get $T$ cell-type-specific *p*-values for each gene $g$ that is tested at this level. We then combine these *p*-values across kernel bandwidths via the Cauchy combination procedure. The Benjamini-Hochberg correction at level $\alpha$ is then applied across all $T$ cell types for each gene. For all gene and index cell-type pairs $(g,i)$ that are significant after correction, we proceed to test whether ${\beta }_{\sigma ,i,n}^{g}=0$ for each niche cell type $n$ again combining *p*-values via the Cauchy combination procedure and applying a Benjamini-Hochberg correction at level $\alpha$ across all niche cell types. For all gene, niche, and index cell-type pairs $(g,i,n)$ that are significant after correction, we conclude that gene $g$ is an $(i,n)$ niche gene.

This hierarchical procedure can be shown to control overall FDR control at approximate level $\alpha (\#Discoveries+\#Families\, Tested )/(\#Discoveries+1)$ [33]. Importantly, this procedure allows the flexibility to report niche effects at the gene and index cell-type levels when, due to multicollinearity caused by colocalizing cell types, it may be difficult to tease apart which cell type in the niche is responsible for the niche-differential expression. Such colocalization induced multicollinearity can be rampant even when the data is at single-cell resolution. In the case where a gene rejects at the cell-type level for cell type$i$, but there is not enough power to identify the niche cell type, we call this gene an $i$-niche gene. Note that we screen for one-sided niche effects by testing the null hypothesis ${\beta }_{\sigma ,i,n}^{g}\ge 0$ and ${\beta }_{\sigma ,i,n}^{g}\le 0$ separately.

### Applying niche-DE over multiple kernel bandwidths

The kernel bandwidth parameter $\sigma$ determines the spatial range of cells that contribute towards the effective niche of the index cell/spot. Because we do not know the optimal $\sigma$ a priori, we perform niche-DE on a grid of $K$ different kernels ${\sigma }_{1},\dots .{\sigma }_{K}$ (Fig. 1B). This gives us gene-level *p*-values $\{{p}_{{\sigma }_{j}}^{g}\}$ for each bandwidth ${\sigma }_{j}$. We also record the log-likelihood score, ${L}_{{\sigma }_{j}}^{g}$, of the negative binomial regression of ${X}_{\left\{s,g\right\}},\mathrm{ on }{N}_{\sigma ,s}$. To pool together *p*-values from each kernel, we use the Cauchy combination test with the weight for ${p}_{{\sigma }_{j}}^{g}$ defined as ${L}_{{\sigma }_{j}}^{g}/(\sum_{i=1}^{K}{L}_{{\sigma }_{i}}^{g})$ to get a kernel-weighted gene-level *p*-value ${p}^{g}$. We apply the BH procedure on the set of kernel-weighted *p*-values ${p}^{g}$ to determine which genes proceed to the cell-type level test. This procedure allows us to test multiple kernel bandwidths, prioritizing bandwidths that give better fit to the data. The same bandwidth-weighting procedure is performed at the cell-type and interaction levels.

### Precision of false-positive control and robustness to spot swapping

**Fig. 2:**
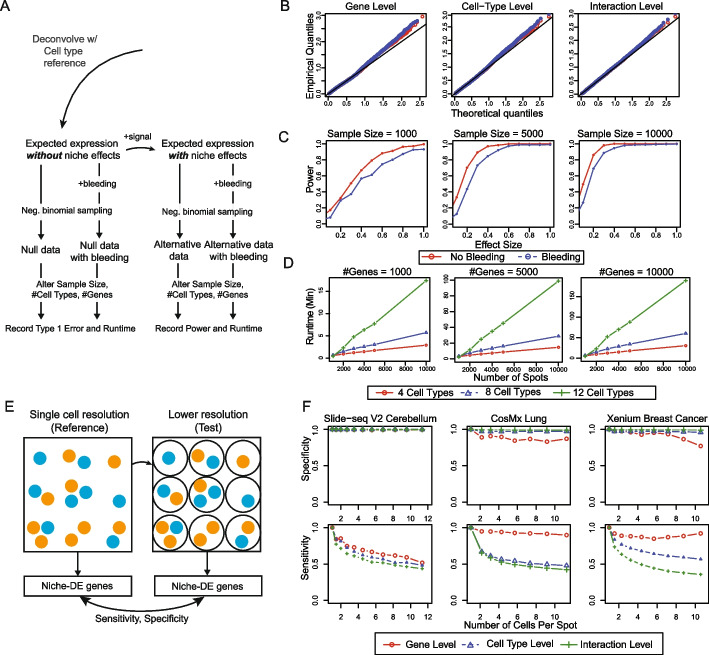
**A** Overview of data simulation: To simulate realistic ST data, we take a real ST dataset and perform deconvolution to calculate the expected expression vector for each spot ${X}_{s}$. To generate data in the absence of niche effects, we simulate expression vectors from a negative binomial distribution with mean ${X}_{s}$ and overdispersion parameter 1. To generate data with niche effects, we specify ${\beta }_{i,n}$ for all index-niche pairs $(i,n)$ and calculate the new expected expression vector for each spot ${Y}_{s}$ based on the niche-DE model. We then simulate expression vectors from a negative binomial distribution with mean ${Y}_{s}$ and overdispersion parameter 1. We also simulate ST data in the presence of spatial bleeding by calculating new expression vectors based on the SpotClean model with local bleeding parameter 0.25. Afterwards, we calculate the type 1 error rate, power, and runtime of niche-DE. **B** Gene level, cell-type level, and interaction-level null *p*-value QQ plots when performing niche-DE on the simulated data. The empirical quantiles are based on those generated by niche-DE on the simulated data. The theoretical quantiles are based on the uniform distribution. **C** Power calculation when performing niche-DE on the simulated data with niche effects of varying sizes. **D** Runtime of nice-DE across the number of genes, the number of cells/spots, and the number of unique cell types present in the data. **E** Pseudo-spot data simulation overview: To simulate spot-level data from single cell level data, we created pseudo spots by partitioning the field of view into equal-sized squares. Counts are aggregated within each square, to create a pseudo-spot. Spot size is defined as the average number of cells in a pseudo-spot. We applied niche-DE to these lower-resolution datasets, and using the niche-DE results from the original high-resolution datasets as the gold standard, we computed the sensitivity and specificity of the niche genes found at each spot size. **F** Gene level, cell-type level, and interaction-level sensitivity and specificity vs spot size in both Slide-seq cerebellum, Xenium breast cancer, and CosMX SMI NSCLC data

First and foremost, we evaluate the accuracy of type I error control for niche-DE testing to ensure that false positives are controlled in the transcriptome-level screen across cell-type configurations. Towards this end, we generated realistic spatial transcriptomic data in the absence of niche-DE genes, as shown in Fig. 2A: Starting from a high-quality Liver mCRC Visium data set matched with scRNA-seq data, we deconvolved each spot $s$ to estimate its cell-type composition vector, which, when multiplied with the reference cell-type-specific gene expression matrix, gave us the expected expression vector for that spot ${\mu }_{s}$. We then sampled a new expression vector ${X}_{s}$ for the spot by drawing from a negative binomial distribution with mean ${\mu }_{s}$ and a given overdispersion parameter. Note that, since expression values are drawn for each spot independently given only the spot’s cell-type decomposition and without influence from its neighbors, data simulated in this way should have no niche-DE genes, that is, ${\beta }_{i,n}^{g}=0$ for all genes $g$ and index-niche cell-type configurations $(i,n)$.

Spot swapping is a known artifact in spatial transcriptomic data, where transcripts from a spot can “bleed” into nearby spots, contaminating the transcript pool of those nearby spots. This can lead to false spatial correlation and, as such, all ST datasets are contaminated to some degree. To examine how spot swapping may affect niche-DE *p*-values, we simulated spatial bleeding according to the SpotClean model where 25% of transcripts are bled into neighboring regions [7]. For each spot $s$, this results in a contaminated mean expression vector ${\mu }_{s}^{c}$. We then sampled a contaminated expression vector ${X}_{s}^{c}$ for the spot by drawing expression values from a negative binomial distribution with mean ${\mu }_{s}^{c}$. We denote by $X=\left\{{X}_{s}\right\}$ and ${X}^{c}=\{{X}_{s}^{c}\}$ the simulated data sets with and without contamination, respectively.

To test the robustness of niche-DE to overdispersion and spot swapping, we perform niche-DE on $X$ and ${X}^{c}$ at the gene, cell-type, and interaction levels. To visualize the *p*-values, we take their negative logarithm. In Fig. 2B, we see that the *quantile-quantile*-plot of niche-DE *p*-values on both $X$ and ${X}^{c}$ are similar to that of a uniform distribution. This indicates that niche-DE effectively controls the type 1 error rate and is robust to spatial bleeding.

To examine if the power of niche-DE across effect sizes and dataset sizes is maintained under spot swapping, for 2000 random genes $${G}_{\beta }$$, we introduced a spike-in effect $${\beta }_{i,n}^{g}$$ that varied within the set $$\{\mathrm{0.2,0.4,0.6,0.8,1}\}$$. For $$g\in {G}_{\beta }$$, let $${\mu }_{s,g,\beta }$$ be the expected expression of gene $$g$$ in spot $$s$$ according to the niche-DE model given $${\beta }_{i,n}^{g}=\beta$$. Similarly, let $${\mu }_{s,g,\beta }^{c}$$ be the expected expression of gene $$g$$ in spot $$s$$ under the spot-swapping model with spike-in effect $$\beta$$. From $${\mu }_{s,g,\beta }$$ and $${\mu }_{s,g,\beta }^{c}$$, we can simulate $${X}_{s,\beta }$$ and $${X}_{s,\beta }^{c}$$ by drawing expression values based on a negative binomial distribution with mean $${\mu }_{s,g,\beta }$$ and $${\mu }_{s,g,\beta }^{c}$$, respectively. To simulate varying dataset sizes (number of spots), we bootstrapped $$B$$ samples from the sets $$\left({N}_{\sigma ,s},{\mu }_{s,\beta }\right)$$ and $$\left({N}_{\sigma ,s}^{c},{\mu }_{s,\beta }^{c}\right)$$ where $$B\in \left\{\mathrm{1000,5000,10,000}\right\},$$$${N}_{\sigma ,s}$$ is the effective niche of spot $$s$$ in the simulated dataset with no spatial bleeding, and $${N}_{\sigma ,s}^{c}$$ is the effective niche of spot $$s$$ in the simulated dataset with spatial bleeding. The results (Fig. 2C) show that there is minimal power loss due to spot swapping.

We believe that the robustness to spot swap is due to the bleeding effect being absorbed into the cell-type proportion estimates during the deconvolution step. Suppose that cell type $i$ in spot $s$ bleeds a proportion $\alpha$ of its transcripts into neighbor spot $s{\prime}$. Perfect deconvolution based on marker genes would infer that spot $s^\prime$ has an additional $\alpha$ cells of type $i$. If a gene $g$ is a cell type $i$ niche gene, it will be bled into neighboring spots more/less than what is expected, resulting in gene $g$ being seen as a cell type $i$ niche gene. Because neighboring cells have similar effective niches, this results in niche-DE detecting similar niche patterns in gene expression. Therefore, contaminated data will have similar niche patterns in gene expression to non-contaminated data. As such, the bleeding leads to biased estimates of cell-type proportions, but the bias is not propagated to the niche-DE estimates.

### Runtime of niche-DE across cell-type complexity, gene set size and number of observations

To benchmark runtime, we performed niche-DE across three different kernel bandwidths in parallel across 4 cores. We then vary the number of cell types, the number of cells/spots, and the number of genes measured. As shown in Fig. 2D, the runtime increases linearly with the number of observations and the gene set size while it increases quadratically with the number of cell types (Fig. 2D). As a reference, it took 1 h to analyze a dataset with 10,000 cells, 10,000 genes, and 8 cell types.

### Recovery of niche-DE genes from ST data at varying spot resolutions

To quantify how much information is lost when using lower-resolution spot- and ROI-based technologies where each spot/region aggregates multiple cells, we simulated spot-level data by aggregating nearby measurements in three publicly available spatial transcriptomic data sets with subcellular resolution: the Slide-seq cerebellum data [38], the Nanostring CosMx SMI non-small cell lung cancer data [29], and the Xenium breast cancer data [12]. To simulate lower-resolution data, we created pseudo spots by partitioning the field of view into equal-sized squares (Fig. 2E). Counts are aggregated within each square. We call the average number of original spots/cells that are contained in each pseudo-spot the ‘spot size’. A larger spot size corresponds to a coarser dataset. We applied niche-DE to these lower-resolution datasets, and, using the niche-DE results from the original high-resolution datasets as the gold standard, we computed the sensitivity and specificity of the niche genes found at each spot size (Fig. 2F).

For the Slide-seq cerebellum data, across all spot sizes, the specificity is maintained at almost exactly 1, indicating that type I error is effectively controlled at all spot resolutions. However, we see a clear trend of sensitivity decreasing as spot size increases. When the spot size is 4 cells, niche-DE recovers about 75% of the gold standard detections, and when the spot size increases to 10 cells, the recall rate drops to 50% at the gene, cell type, and interaction levels. This trend may be also be in part due to sample size decrease, since, when the spot size is 10, the corresponding coarser dataset has 10 times less data points.

For the CosMx SMI non-small cell lung cancer data as well as the Xenium breast cancer data, we see a similar trend: Across all spot sizes, specificity is maintained at almost exactly 1 for the cell type and interaction-level tests, and above 90% for the gene-level tests. Sensitivity decreases, as expected, with increasing spot size. For this data set, recall rate at the gene level is maintained above 90% even at spot size of 10 cells. However, as for the Slide-seq cerebellum data, recall rates for the cell-type- and interaction-level tests drop substantially as spot size increases, leveling at 50% when the spot size is 10. Unlike the slide-seq data, the drop-off in sensitivity is sharper when spot size increases from 1 to 2. We believe that this is due to the larger number of cell types in this data set (20/18 versus 8), and thus the number of niche-index interactions tested across colocalizing cell types makes it hard to find exactly which index-niche pair(s) are driving the significance of a niche-DE gene.

These results indicate that, while there is indeed loss of power with decreased spatial resolution, over half the signals detectable at the subcellular resolution can be detected by niche-DE at a spot size of 10 cells, without increasing false-positive rate.

### Inferring ligand-receptor interactions via niche-DE

**Fig. 3:**
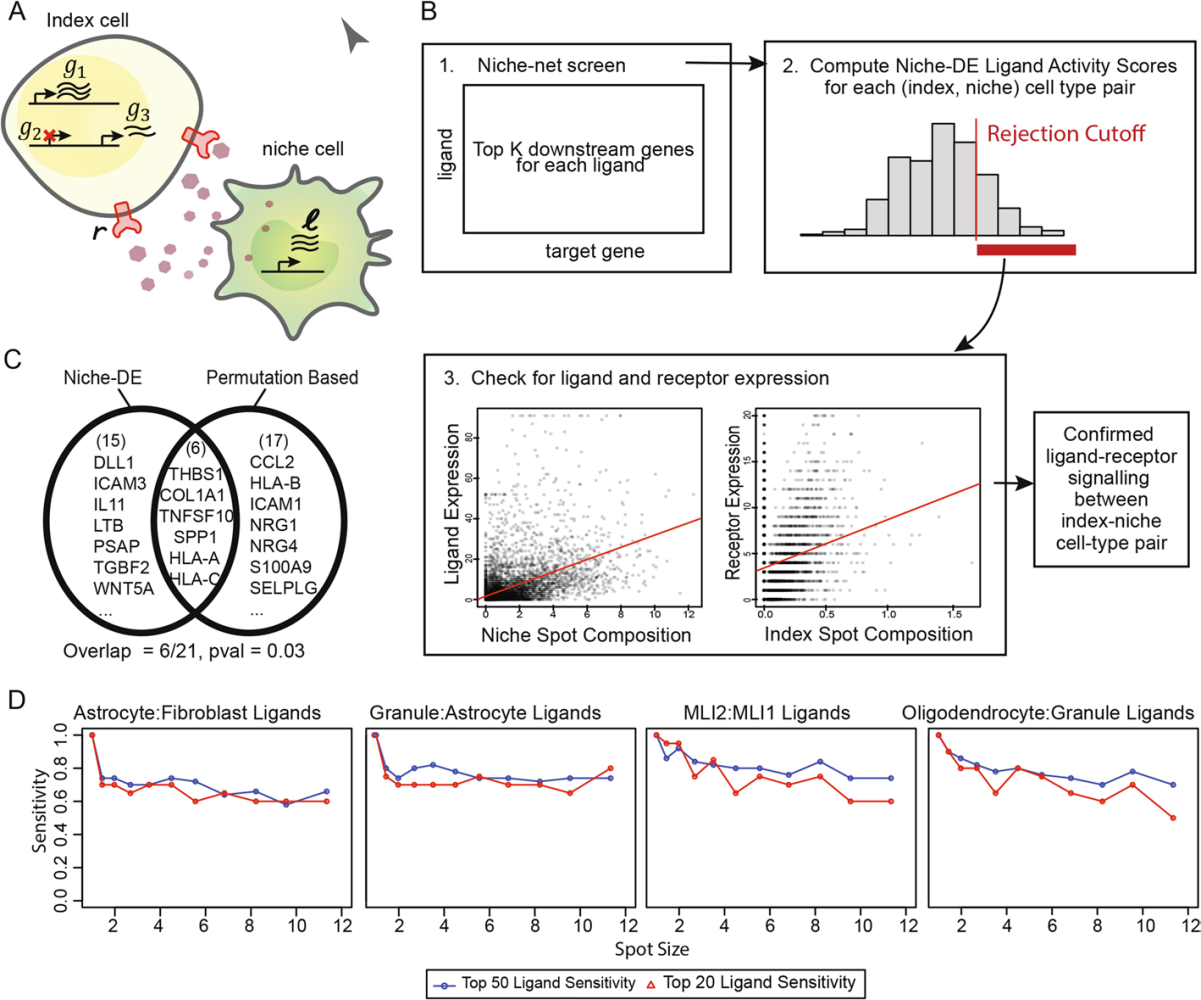
**A** Ligands from the niche cell type are received by the index cell type, resulting in a change in downstream gene expression. We expect these downstream genes to be $(index,niche)$ genes. **B** Overview of niche-DE Ligand-Receptor pipeline: We aim to determine which ligand-receptor pairs are active between the ligand expressing niche cell type $n$ and the receptor expressing index cell type $i$. (1): Using the ligand-target potential matrix from niche-net, we extract the top $K$ downstream genes for each ligand. (2): Using these downstream genes, we calculate a ligand activity score using the niche-DE T-statistic vector between index-niche pair $\left(i,n\right)$ for the top $K$ downstream genes. (3): Ligands with an activity score greater than a threshold value and their corresponding receptor(s) are screened for expression in the niche cell type. **C** Comparison of ligands inferred between the niche-DE-based pipeline and a permutation-based pipeline [29]. **D** Sensitivity of top 20 and top 50 ligands by ligand activity score vs spot size using slide-seq cerebellum data as the reference

To determine which extracellular signaling mechanisms are driving niche-DE patterns between index cell type $i$ and niche cell type $n$, we developed a procedure integrating Niche-differential genes, ligand expression, receptor expression, and Niche-Net [27] data which links ligands with downstream target genes (Fig. 3A). The procedure, illustrated in Fig. 3B, starts with the ligand-target matrix $A=\{{A}_{l,g}:l=1,\dots ,L;g=1,\dots ,G\}$ obtained from Niche-Net, where $L$ is a set of ligands and $G$ is a set of target genes. ${A}_{l,g}$ reflects the confidence that ligand $l$ can regulate the downstream expression of gene $g$. Complementing this, for index cell type $i$, niche cell type $n$, and kernel bandwidth $\sigma$, Niche-DE provides a $G$ dimensional vector of one-sided *t*-statistics ${B}_{\sigma ,i,n}=\{{B}_{\sigma ,i,n,g}:g=1,\dots ,G\}$, where ${B}_{\sigma ,i,n,g}$ reflects whether or not gene $g$ is an ${\left(i,n\right)}^{+}$ niche gene operating at kernel bandwidth $\sigma$. For each ligand $l$, we first identify the top downstream genes using $\{{A}_{l,g}\}$ (Fig. 3B step 1), and then calculate a ligand activity score for each niche and index cell type pair $(i,n)$, which measures the degree to which these downstream targets are found to be niche-associated between $i$ and $n$ (Fig. 3B step 2). The null distribution of this activity score can be determined, and the ligands whose activity score pass the null *p*-value threshold are assumed to be the most likely ligands expressed by niche cell type $n$ in its interaction with index cell type $i$. We call this set of ligands ${C}_{i,n}$.

Ligands in the candidate ligand set ${C}_{i,n}$ should be expressed by the niche cell type $n$, but this obvious condition has not yet been checked. Checking this condition is especially important since ligands may share similar downstream target genes, and thus, a ligand may have spuriously high activity scores due to lack of specificity in its Niche-net profile. Therefore, for each $l\in {C}_{i,n}$, we perform a statistical test to confirm that the ligand is indeed expressed in the niche cell type $n$, and that at least one of its known receptors is indeed expressed in the index cell type $i$ (Fig. 3B step 3). We call this combined approach niche-LR, for niche-ligand-receptor analysis. Details of niche-LR, including the computation of ${C}_{i,n}$ and the follow-up statistical test for niche expression of the ligand and index expression of the receptor, are described in “Methods”.

On the CosMx SMI NSCLC data [29], a permutation-based approach was proposed to assess ligand-receptor co-expression between adjacent cell pairs. We compare the Niche-LR detected ligand-receptor pairs to those detected by the permutation-based method, see Additional file 1: Tables S1 and S2. Consider, for example, signaling between memory CD8 T (index cell type) and tumor (niche cell type). Out of the 21 unique ligands belonging to ligand-receptor pairs identified by Niche-LR, 6 ligands overlap with those identified by the permutation-based method (*p*-value = 0.03, Fig. 3C). The overlap (*THBS1, COL1A1, TNFSF10, SPP1, HLA-A,* and *HLA-C*) is significant; however, the lack of overlap is also not surprising, given that the two approaches differ substantially: The permutation-based procedure, which is designed specifically for spatial transcriptomic data at single-cell resolution, ignores the expression of genes downstream of the ligand, and, by explicitly focusing on adjacent cells, ignores signaling between cells that are not immediate neighbors. Thus, genes downstream of the ligand does not need to show evidence of niche-DE for the ligand to be detected by [11]. However, [11] is expected to miss ligand-receptor channels that operate over larger distances, as well as those where RNA-level expression does not closely track the colocalization between the niche and index cell types.

To assess the accuracy of niche-LR at varying spot resolutions, we applied the method to the original and spot-aggregated datasets created as described in Fig. 2E. The results (Fig. 3D) show that the top 20 and 50 candidate ligands in the Slide-seq cerebellum data are generally conserved across spot sizes and index-niche cell-type configurations. In the CosMx SMI lung cancer data, there is larger drop-off in overlap. We believe that this is due to the large number of cell types being screened (22 cell types leading to 484 index-niche configurations versus 8 cell types leading to 64 index-niche configurations for cerebellum), thus leading to higher numbers of colinear covariates in the regression and a vastly expanded number of parallel tests, decreasing the sensitivity for specific cell-type configurations. Due to the small gene panel used in Xenium, niche-LR was not performed.

### Niche-DE detects tumor-fibroblast niche interactions with validation by CODEX imaging in liver metastases of colorectal carcinoma

We applied niche-DE and niche-LR to the integrative analysis of five 10X Visium samples obtained from liver metastases of colorectal carcinoma. For cell-type deconvolution and mean expression profiles, we based our analysis on the reference scrna-seq data from a previous study from Sathe et al. [39]. Three of the samples (patients 1–3) come from another study on liver metastasis by Wu et al. [40], while two additional samples (patients 4–5) were generated in this study. CODEX data has been generated for patient 4. Additional file 2: Table S1 gives the details, including quality metrics, of these data sets. We deconvolved each sample using the single-cell reference data set from Sathe et al., yielding the proportions of each of the 6 major cell types (hepatocytes, epithelial, fibroblast, macrophage, T lymphocytes, B lymphocytes) in each spot. Spatial maps of the estimated cell-type proportions are shown in Additional file 3: Fig. S1.

**Fig. 4:**
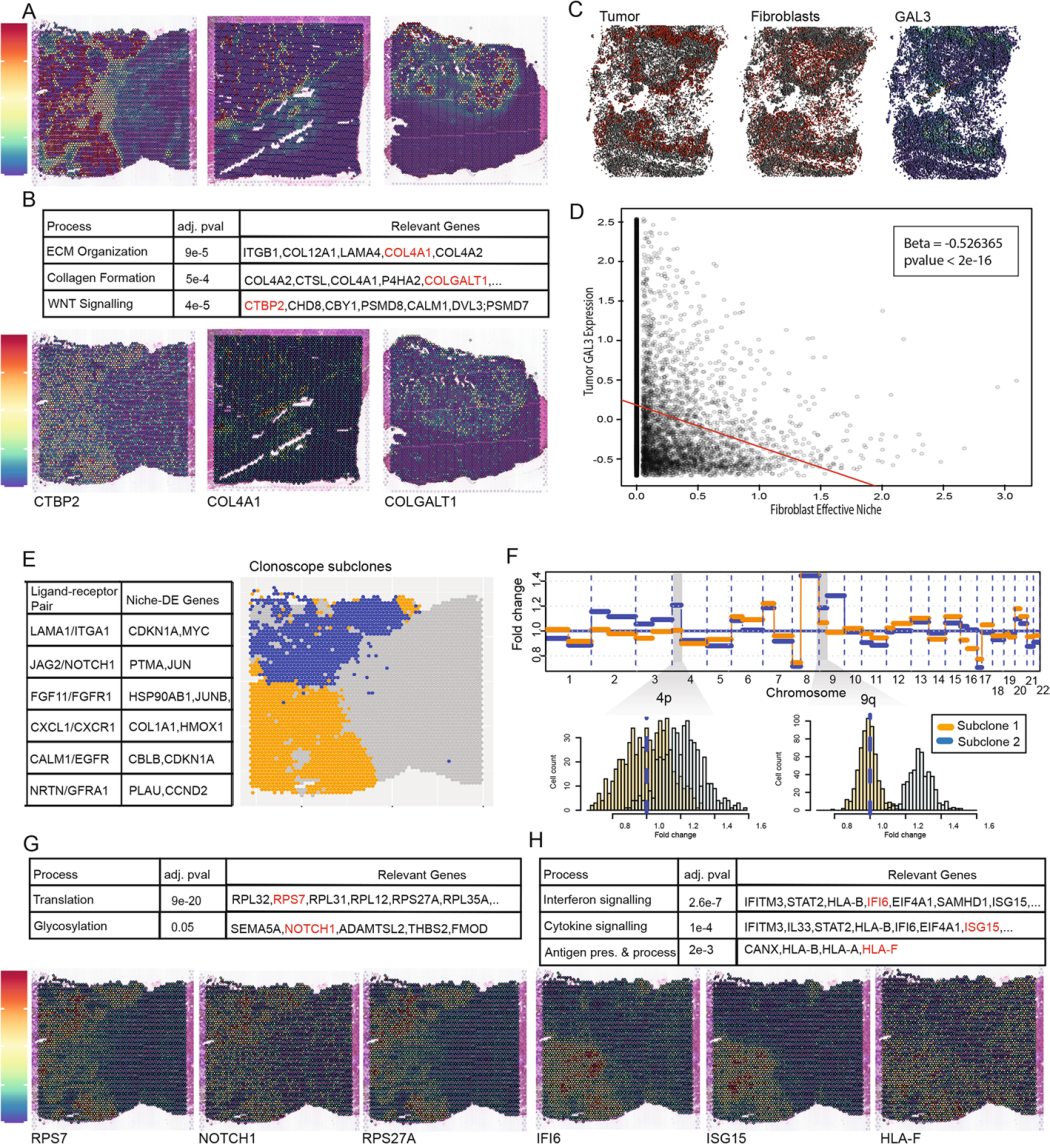
**A** Colocalization heatmaps between fibroblasts and tumor in liver samples 1, 2, and 3. **B** Using niche-DE marker genes in fibroblasts near tumor as input, pathway enrichment analysis finds ECM organization, collagen formation, and WNT signaling as top processes in fibroblasts in the presence of tumor cells. Spatial heatmaps of CTBP2*, COL4A1*, and *COL1GALT1* confirm expression of pathway-related genes in the presence of tumor. **C** Spatial heatmap of tumor abundance, fibroblast abundance, and GAL3 expression in CODEX data of liver metastasis of colorectal cancer in patient 4. **D** Regression of tumor GAL3 expression on their fibroblast effective niche in CODEX data finds a significantly negative coefficient consistent with those found by niche-DE. **E** Ligand-receptor pairs found between fibroblasts and tumor via niche-LR include *LAMA1/ITGA1*, *JAG2/NOTCH1*, and *CXCL1/CXCR1*. **F** Clonalscope [41] finds two major tumor subclones in liver sample 1. Analysis finds an amplification of chromosomes 4p and 9q in subclone 2 relative to subclone 1. **G** Using niche-DE marker genes in fibroblasts near subclone 2 relative to subclone 1 as input, pathway analysis finds translation and glycosylation among enriched processes. Spatial heatmaps of *RPS7*, *NOTCH1*, and *RPS27A* confirm differential expression of pathway-related genes in the region of the tissue containing tumor subclone 2. **H** Using niche-DE marker genes in fibroblasts near subclone 1 relative to subclone 2 as input, pathway analysis finds interferon signaling, cytokine signaling, and antigen presentation among enriched processes. Spatial heatmaps of *IFI6*, *ISG15*, and *HLA-F* confirm differential expression of pathway-related genes in the region of the tissue containing tumor subclone 1

Since fibroblasts make up a substantial fraction of the cells in all samples, and since cancer-associated fibroblasts are an emerging target for anti-cancer therapy, we first focus on the niche interactions between fibroblasts and tumor cells. At FDR threshold of 0.05, niche-DE found 1117 genes whose expression in fibroblasts are significantly associated with the niche-enrichment of tumor cells. These genes are given in Additional file 4: Table S1. Pathway enrichment analysis of ${\left(fibroblast,tumor\right)}^{+}$ genes identifies extracellular matrix organization, collagen production, and WNT signaling as the top three processes (Fig. 4B). This finding concurs with recent findings that collagen production and extracellular matrix remodeling are two of the most pronounced properties of tumor-associated fibroblasts versus their normal counterparts [39]. A full list of enriched pathways is given in in Additional file 4: Table S2. A visual check of the spatial distribution for three of the niche-DE genes (*COLGALT1, COL4A1*, and *CTBP2*), each in a separate patient sample, reveals that niche-DE gene expression are indeed enriched in regions of the tissue where fibroblasts and tumor cells colocalize (Fig. 4B). Such expression enrichment in regions of index-niche cell-type colocalization is not always the case with niche-DE genes, as the niche-DE model uses flexible kernel bandwidths to allow for interactions beyond those between immediate neighbors.

We performed Ligand-Receptor analysis using niche-LR, detecting 25 unique ligands from tumor cells among the confirmed ligand-receptors between fibroblasts and tumor. This includes Laminin family genes *LAMB1, LAMC1* which can bind to integrin family receptors on fibroblasts. Integrin signaling has been demonstrated to control fibroblast activation and response to mechanical signals in the TME [42]. Other ligand-receptor channels included *JAG2/NOTCH1,FGF11/FGFR1,CXCL1/CXCR1,CALM1/EGFR*, and *NRTN/GRFA1* (Fig. 4E). The full list of ligand-receptor interactions identified from the data can be found in in Additional file 4: Table S3.

We also applied Niche-DE to identify genes whose expression in tumor cells are niche-associated with the enrichment of fibroblasts, with results shown in Additional file 5: Table S1. Among the top hits in patient 4 is *GAL3*, whose expression in tumor cells was found to be negatively associated with niche-enrichment of fibroblasts. Following-up on this finding, we analyzed the CODEX sample of patient 4. The CODEX protein panel, which includes *GAL3*, is in Additional file 5: Table S2. Niche-DE analysis at the single-cell level in CODEX detects a similar pattern of under expression of *GAL3* in tumor cells with increasing effective niche presence of fibroblasts (Fig. 4C). A regression of tumor cell *GAL3* expression on the effective niche fibroblast concentration gives a slope of −0.53 (*p*-value < 2^−16^, Fig. 4D).

For comparison, we also applied C-Side to the Visium sample from patient 1. Since C-Side requires the user to input the covariate matrix, and does not give instructions on what to use, we used as input the effective niche computed by niche-DE. C-Side does not allow combining evidence across multiple neighborhood sizes, and thus we used a fixed kernel width of 250 pixels. Next, we extracted the niche-specific coefficients and standard errors from C-Side to manually compute their *p*-values and perform FDR control at the 0.05 level via Benjamini-Hochberg procedures, giving us 3926 upregulated and 1815 downregulated interaction-level signals. Niche-DE gives 5676 upregulated and 3861 downregulated interaction-level signals. The Venn diagram of discoveries made between the two methods can be found in Additional file 6: Fig. S1. Even when the two methods both start with the covariate matrix computed by niche-DE, there are significant differences, which we believe is due partly to the differences discussed above (i.e., niche-DE adjusts for cell type-specific library size and conducts hierarchical FDR control, which is not performed in C-Side).

An inspection of (fibroblast,tumor)+ genes found by niche-DE and C-Side shows that C-Side finds 167 genes and niche-DE find 319 genes with 57 genes overlapping between both gene sets. Pathways associated with genes found by C-Side but not niche-DE correspond to metabolism whereas pathways associated with genes found by niche-DE but not C-Side correspond to translation, extracelluar matrix organization, collagen formation, and metabolism. Fifty genes found by niche-DE but not C-Side code for ribosomal proteins, which should be upregulated in fibroblasts undergoing tumor-associated extracellular matrix remodelling.

### Niche-DE identifies subclone-specific niche interactions between tumor cells and fibroblasts

Tumor cells exhibit a large degree of heterogeneity due to subclonal evolution driven by both intrinsic genomic and epigenomic instability as well as extrinsic factors in its microenvironment. Thus, identifying different subclones of tumor cells in situ, and comparing the ways that the subclones interact with their local niche, may allow us to learn about mechanisms of tumor growth and invasion. Using Clonalscope [41], we identified 2 spatially distinct tumor subclones in Liver Sample 1. As shown in Fig. 4F, subclone 1 is located predominantly at the lower left region of the slide, while subclone 2 is located predominantly at the upper left region of the slide(Fig. 4E). These two subclones are distinguished by chromosome 4p and 9q amplification in subclone 2 (Fig. 4F). The histopathology of the liver sample shows a conventional gland-forming adenocarcinoma. The region containing subclone 2 has less desmoplastic stroma, more architectural complexity, and some medullary-like features such as foci of a solid growth pattern compared to the region containing tumor subclone 1. Due to the more irregular and decreased gland formation, tumor subclone 2 is less differentiated relative to tumor subclone 1. Applying Niche-DE to this sample, with fibroblasts as the index cell type and each of the two subclones as niche cell types, we detect niche-differential expression that is specific to each subclone. Reactome analysis of marker genes of fibroblasts near subclone 1 reveals enrichment of interferon and cytokine signaling. Applying Niche-DE with subclone 1 as index cell type and fibroblasts as niche cell type identifies IFNGR1 to be a ${\left(subclone 1, fibroblast\right)}^{+}$ niche gene as well. Reactome analysis of marker genes of fibroblasts near subclone 2 reveals enrichment of *glycosylation* and general translation to be enriched processes. These significant differences between fibroblasts that infiltrate distinct subclones at such close spatial proximity suggest that cancer-associated fibroblasts are highly plastic in adapting to subtle changes in their local tumor microenvironment. The full list of marker genes for each subclone can be found in Additional file 7: Tables S1 and S2. The full list of enriched pathways associated with each index-niche cell-type configuration can be found in Additional file 7: Tables S3 and S4. An annotation of the high-resolution H&E image for liver tissue can be found in Additional file 8: Fig. S1.

### Niche-DE analysis identifies marker genes and signaling mechanisms specific to tumor-associated macrophages in liver metastasis

**Fig. 5:**
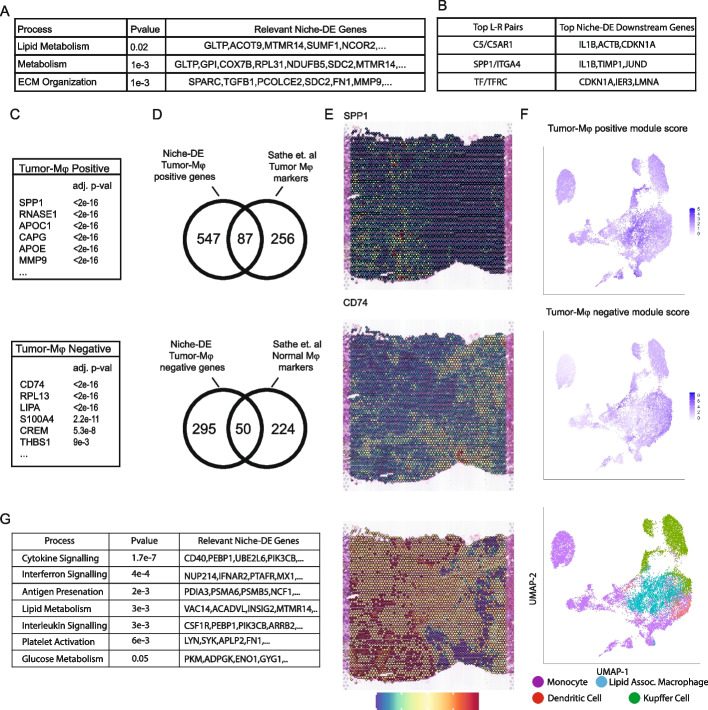
**A** Using niche-DE marker genes in macrophages near tumor as input, pathway enrichment analysis finds ECM organization, metabolism, and lipid metabolism as top processes in macrophages in the presence of tumor cells. **B** Ligand-receptor pairs found between macrophages and tumor via niche-LR include *C5/C5AR1*, *SPP1/ITGA4*, and *TF/TFRC.***C** Lists of niche-DE-positive and negative tumor-associated macrophage marker genes. **D** Overlap of niche-DE marker genes with lists found by Sathe et al. [39]. **E** From top to bottom. Top Two: Spatial heatmaps of *SPP1* and *CD74* confirm differential expression of marker genes in the region of the tissue containing tumor and hepatocytes respectively. Bottom: Differential spatial colocalization plots of macrophages near tumor and macrophages near hepatocytes. Larger values shown in red correspond to regions that contain macrophages near tumor and smaller values shown in blue correspond to regions that contain macrophages near hepatocytes. Regions in yellow correspond to regions with no macrophages or regions with macrophages that have a similar amount of tumor cells and hepatocytes in their effective niche. **F** From top to bottom. Top Two: Feature plot of module scores for the set of niche-DE tumor-associated macrophage markers finds enrichment in the region of the UMAP corresponding to lipid-associated macrophages. Feature plot of module scores for the set of niche-DE normal macrophage markers finds depletion in the region of the UMAP corresponding to lipid-associated macrophages. Bottom: UMAP of scrna-seq from Wu et al. [40]. Cells were filtered to only include macrophages, monocytes, and dendritic cells. **G** Using niche-DE marker genes in macrophages near tumor relative to hepatocytes as input, pathway analysis finds interferon signaling, cytokine signaling, and lipid metabolism among enriched processes

We continue the integrative analysis of the Visium liver metastasis samples depicted in Fig. 4 and Additional file 3: Fig. S1. Reactome pathway analysis of (macrophage,tumor)+ genes identified by niche-DE revealed a significant enrichment of processes related to extracellular matrix (ECM) organization and metabolism (Fig. 5A). Ligand-receptor analysis between macrophages and tumor cells identify *C5/C5AR1,SPP1/ITGA4*, and *TF/TFRC* as the ligand-receptor signaling mechanism driving these niche-DE genes (Fig. 5B). Full lists of confirmed ligand-receptor pairs, (macrophage,tumor)+ genes, and pathway analysis results can be found in Additional file 9: Tables S3, S4 and S5.

The liver microenvironment contains inflammatory macrophages derived from monocytes as well as non-inflammatory tissue-resident macrophages such as Kupffer cells [43]. Tumor-associated macrophages (TAM) represent a further reprogrammed macrophage subtype in the metastatic TME. Identifying and characterizing these cell states using only scRNA-seq is challenging given the similarity of their transcriptional profiles. However, in spatial transcriptomic data, we expect these two cell populations to be niche-differentiable as macrophages from the normal liver should reside near hepatocytes while TAMs should be enriched near tumor cells. We developed an extension of the Niche-DE procedure to not only identify niche-DE genes, but to contrast the degree of niche-DE between two different niche configurations for the same index cell type. For example, we define TAM marker genes as genes whose expression in macrophages have significant positive association with tumor cell concentration in niche, but insignificant or negative association with hepatocyte concentration in niche. We define normal macrophage marker genes as the converse. Using this approach, at FDR threshold of 0.05 we identified 345 genes, including *CD74, LIPA*, and *CREM* as niche-DE marker genes of normal liver macrophages (Fig. 5C). These genes have been previously shown to be enriched in Kupffer cells from normal liver [21], thus validating this result. Niche-DE TAM marker genes included *APOC1, APOE, SPP1*, and genes from the MMP family (Fig. 5C). Eighty-seven out of 634 niche-DE TAM marker genes also overlapped with previously identified scar-associated macrophages [44] (Fig. 5D). The association of SPP1 upregulation with TAMs also confirms our previous finding, made using CODEX, of a *SPP1*+ pro-fibrogenic TAM cell state in liver metastases [39], and the finding of a *SPP1*+ TAM cell state in primary colorectal carcinoma [45]. Spatial heatmaps of *SPP1* and *CD163* confirm the localization of the expression of these genes in, respectively, the tumor and healthy regions of the tissue (Fig. 5E). Full lists of niche-DE TAM and normal macrophage marker genes can be found in Additional file 9: Tables S1 and S2.

To further follow-up on these results, we obtained single-cell RNA-seq data from paired samples of colorectal cancer and adjacent colon, liver metastasis and adjacent liver, lymph nodes along colon, and peripheral blood mononuclear cells (PBMC) [40]. On this data set, we computed module scores for the set of niche-DE tumor-associated macrophage markers and normal-associated macrophage markers identified above from the VISIUM data. We performed cell-type classification via label transfer [31] from a liver cell atlas [46], and after subsequent denoising [35], compared the niche-DE module scores to the transferred cell-type labels (Fig. 5F). For cells classified as lipid-associated macrophages in the liver cell atlas, the niche-DE tumor-associated macrophage module score is high, while the niche-DE normal macrophage module score is low. In contrast, for cells labeled as Kupffer cells, the niche-DE normal macrophage module score is high, while the niche-DE tumor-associated macrophage module score is low. Reactome analysis of niche-DE tumor-associated macrophage marker genes finds glucose and lipid metabolism, as well as HDL-mediated lipid transport as significantly enriched processes (Fig. 5G). A full list of enriched pathways can be found in Additional file 9: Table S6. These results are concordant with previous findings that metabolic reprogramming, and in particular lipid metabolic reprogramming, is a key factor in the regulation of TAM differentiation, polarization, and anti-tumor responses [47].

### Niche-DE analysis identifies signalling mechanisms between fibroblasts and proximal tubular cells in kidney fibrosis

As another example, we applied niche-DE to the analysis of two 10X Visium samples from chronic kidney disease (CKD) [48]. The human kidney is an organ with rich architectural complexity, composed of distinct spatial domains with specialized kidney-specific cell types such as proximal tubular cells interacting with immune cells and fibroblasts. Chronic kidney disease (CKD) is characterized by excessive production and deposition of extracellular matrix proteins, leading to scarring in the tissue, fibrosis, and impairment of renal function [49]. Of the two samples we analyzed, one came from a donor with hypertensive kidney disease (fibrosis 60%), and the other from a donor with diabetic kidney disease (fibrosis 30%). Basic quality metrics of the Visium data are given in Abedini et al. [48].

**Fig. 6:**
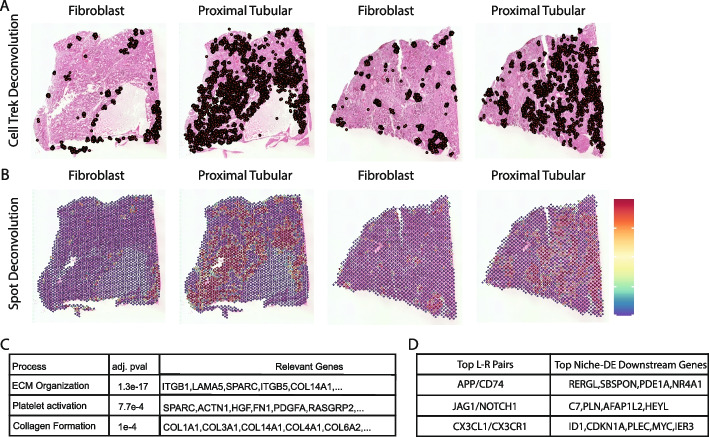
**A** Deconvolution results for fibroblasts and proximal tubular cells in two Visium kidney fibrosis samples using CellTrek [50]. CellTrek maps single cells to spatial locations through coembedding and metric learning approaches. **B** Spot-level deconvolution results for fibroblasts and proximal tubular cells. Using the single-cell level deconvolution with CellTrek, we aggregate to achieve spot-level deconvolution. **C** In line with previous studies [49], pathway enrichment analysis using niche-DE marker genes in fibroblasts near proximal tubular cells as input finds ECM organization, platelet activation, and collagen formation as top processes. **D** Ligand-receptor pairs found between fibroblasts and proximal tubular cells via niche-LR include APP*/CD74, JAG1/NOTCH1,* and *CX3CL1/CX3CR1*. In particular NOTCH signaling is known to be induced in fibrosis [49]

Cell-type deconvolution was performed using CellTrek (Fig. 6A), which maps cells from a reference single-cell atlas to the Visium tissue slice [50]. The two Visium samples have tissue matched single-cell RNA-seq data, which were used to derive accurate estimates of cell-type proportions. Spot-level cell-type compositions were computed from the CellTrek results by dividing the number of cells of the given cell type mapped to a spot by the total number of cells mapped to that spot (Fig. 6B). Niche-DE was applied using the original spot-level expression vectors and the CellTrek-based cell-type proportion estimates as inputs.

While CKD is a complex disease that involves many cell types, much attention have focused on the role of fibroblasts, the source of the extracellular matrix, and proximal tubule cells, the “target” of renal injury. Thus, we will focus our analysis on these two cell types. Controlling the FDR at 0.05, we find 456 genes upregulated in fibroblasts near proximal tubular cells and 370 genes upregulated in proximal tubular cells near fibroblasts.

A reactome pathway analysis of (fibroblast, proximal tubular)+ genes identified by niche-DE revealed that fibroblasts see a significantly increased expression of processes related to extracellular matrix (ECM) organization and collagen formation (Fig. 6C) associated with proximal tubule cells in its niche. Ligand-receptor analysis of these (fibroblasts, proximal tubular)+ genes identify *JAG1/NOTCH1, APP/CD74*, and *CX3CL1/CX3CR1* as the ligand-receptor signaling mechanism driving these niche-DE genes (Fig. 6D). In particular NOTCH signaling is known to be reinduced during fibrosis [49]. Analysis of (fibroblast, proximal tubular)+ genes also reveal that myofibroblast marker genes such as *POSTN* and *FN1* [49] are upregulated in fibroblasts. In proximal tubules, niche-DE identifies *VCAM-1* as upregulated in the presence of fibroblasts. VCAM-1 is a profibrotic and proinflammatory protein expressed by proximal tubule cells during injury [49] and is a key marker of injured proximal tubule cells. The table of (fibroblast,proximal tubular)+ genes confirmed ligand-receptor pairs between fibroblasts and proximal tubular cells, pathway analysis, and the table of (proximal tubular,fibroblast)+ genes results can be found in Additional file 10: Tables S1, S2, S3 and S4. These results show that niche-DE is able to recover relevant signals in a scenario with high cell-type diversity and architectural complexity such as chronic kidney disease.

## Discussion

For spot-level spatial transcriptomic data, an important pre-processing step prior to performing niche-DE is deconvolution to estimate the cell-type proportions within each spot. The accuracy by which niche-DE can detect cell-type-specific niche-DE genes relies on the accuracy by which those cell types can be distinguished. Matched single-cell data is not required for deconvolution as a single-cell reference atlas of the same tissue can be used to perform deconvolution in most cases. This was illustrated in our analysis of the liver metastasis data, for which there is no matched single-cell RNA sequencing data.

A central factor in the sensitivity of niche-DE for detecting niche genes between a given cell-type pair is its rate of co-occurrence. If a cell type is rare in the spatial sample, then niche-DE is unlikely to find any signal due to insufficient sample size. Niche-DE will also be under-powered if two cell types never colocalize or always colocalize. We need to observe the two cell types both together and far apart, the latter providing the contrast for differential expression. For spot-level data, a simple rule that we recommend is that there be at least 30 spots that contain the niche cell type and have the index cell type in its niche.

Currently, two of the most common analyses of spatial transcriptomic data are spatially variable gene analysis and cell-type colocalization analysis. In the “Background”, we expounded on the differences between these analyses. We stress here that the conclusions made from a niche-DE analysis do not subsume that of a spatially variable gene analysis or a cell-cell interaction analysis. A gene that is an ${\left(i,n\right)}^{+}$ niche gene may not be a spatially variable gene if the index and the niche cell type do not colocalize according to some spatial pattern. Further, the index cell and niche cell do not need to colocalize more than random in order to have niche-DE genes between them. Therefore, we recommend that niche-DE be used in conjunction with, not as an alternative to, current spatial analyses.

Among existing methods, the method that is most similar to niche-DE in terms of analysis goal is C-Side [28]. However, there are important differences, as highlighted under “Results”. We want to note here that in addition to differences in the model, niche-DE allows for automatic consideration of multiple gene-specific kernel bandwidths and integrated hierarchical FDR control. Since we do not know the optimal bandwidth size a priori, we must test multiple bandwidths and combine the results to come up with a single *p*-value. Analysis of multiple bandwidths and the corresponding *p*-value combination step is automatic in niche-DE whereas this must be done manually in C-side. Since the number of hypotheses scales linearly in the number of genes and quadratically in the number of cell types, the number of hypothesis tests can easily exceed 1 million limiting the power of traditional multiple testing approaches. These differences lead niche-DE to detect more relevant genes than C-side, as shown on the liver metastasis example, even when the effective niche matrix computed by niche-DE is used as input to C-side.

A limitation of niche-DE is that it only considers two-way interactions, e.g., differential expression of macrophages genes near fibroblasts. This may be too simplistic for spatial transcriptomic datasets where two colocalizing cell types can be found in multiple global environments, e.g., macrophages near fibroblasts in tumor regions and macrophages near fibroblasts in healthy regions. One way to allow for interactions of more than 2 cell types is to augment the effective niche vector in niche-DE via the inclusion of all pairwise products between cell types. However, the number of hypotheses increases exponentially with the order of interaction, and although feasible, we believe the current data size and quality only allow sensitive detection of pairwise interactions.

## Conclusions

We have presented a new method, niche-DE, for identifying cell type-specific genes whose expression is significantly up- or downregulated in the context of specific spatial niches. Niche-DE lends easily to integrative analysis, has robust type 1 error control, and allows for multiple kernel bandwidths for effective niche calculation. Through benchmarking studies conducted using single-cell and subcellular resolution spatial transcriptomic data, we showed that applying niche-DE to lower-resolution spot-level data reliably recovers cell-cell interactions observed at the single cell level. The output of niche-DE can be used in a variety of ways, including but not limited to pathway enrichment analysis, ligand-receptor analysis, and niche marker gene analysis. In particular, we developed a procedure that integrates niche-DE statistics and niche-net ligand-target matrices to infer putative ligand-receptor signaling channels that underlie niche-differential gene expression. We illustrate the effectiveness of niche-DE through an integrative analysis of 10X Visium samples of liver colorectal cancer metastasis and chronic kidney disease. In the liver metastasis samples, we corroborate our findings though parallel analyses of single-cell RNA sequencing (scRNA-seq) and CODEX data for the same tissue type. In the kidney fibrosis example, we show that we can uncover well-known disease-associated genes.

## Methods

### Derivation of the spot-level niche-DE model

Model (1) specifies a log-linear dependence of expected gene expression on niche-composition. If the dependence were linear, then we could simply sum across all cells within each spot to derive an equivalent spot-level model with the same interpretations for the parameters ${\beta }_{\sigma ,{t}_{i},t\mathrm{^\prime}}^{g}$. However, due to the gross variance inflation in gene expression data, it is necessary for the regression to be in log space. Here, we first show that the mean relationship in the single-cell-level model can be approximated by a linear model, and then use that approximation to derive the equivalent spot-level regression formula. Starting with Eq. (1),

$$\sum_{n=1}^{T}{N}_{\sigma ,i,n}{\beta }_{\sigma ,{t}_{i},n}^{g}={\text{log}}\left(\frac{{\mathbb{E}}\left[{Y}_{i,g}|{T}_{i},{N}_{\sigma ,i}\right]}{{\mu }_{{T}_{i},g}}\right)={\text{log}}\left(1+\frac{{\mathbb{E}}\left[{Y}_{i,g}|{T}_{i},{N}_{\sigma ,i}\right]-{\mu }_{{T}_{i},g}}{{\mu }_{{T}_{i},g}}\right).$$

Using the approximation ${\text{log}}\left(1+x\right)\approx x$ for $x\to 0$, and assuming that the overall effect size ${\mu }_{{T}_{i},g}^{-1}\left\{{\mathbb{E}}\left[{Y}_{i,g}|{T}_{i},{N}_{\sigma ,i}\right]-{\mu }_{{T}_{i},g}\right\}$ is small, the above leads to the approximation

$$\frac{{\mathbb{E}}\left[{Y}_{i,g}|{T}_{i},{N}_{\sigma ,i}\right]-{\mu }_{{T}_{i},g}}{{\mu }_{{T}_{i},g}}\approx \sum_{n=1}^{T}{N}_{\sigma ,i,n}{\beta }_{\sigma ,{t}_{i},n}^{g}$$

and thus,

3 $${\mathbb{E}}\left[{Y}_{i,g}|{T}_{i},{N}_{\sigma ,i}\right]\approx {\mu }_{{T}_{i},g}+{\mu }_{{T}_{i},g}\sum_{n=1}^{T}{N}_{\sigma ,i,n}{\beta }_{\sigma ,{t}_{i},{\text{n}}}^{g}$$

Hence, the cell-level log-linear model can be approximated, at the mean level, by a linear model, but with the extra ${\mu }_{{T}_{i},g}$ factor multiplied to the effect size for each cell.

Now consider spot-level data. Let $s\left(i\right)$ denote the spot where cell $i$ resides and ${X}_{s,g}$ be the observed expression of gene $g$ in spot $s$. Using the same kernel function, we compute an effective niche for each spot $s$, which approximates the effective niche for each cell $i$ where $s\left(i\right)=s$. Since spots can contain a multitude of cell types, let ${n}_{s}$ be the cell-type composition vector for spot $s$, i.e., ${n}_{s}=\left({n}_{s,t}:t=1,\dots T\right),$ where ${n}_{s,t}$ is the number of cells of type $t$ in spot $s$. We sum the approximations (3) across all cells within each spot to yield

$${\mathbb{E}}\left[{X}_{s,g}|{n}_{s,t},{N}_{\sigma ,s}\right]=\sum_{i:s\left(i\right)=s}{\mathbb{E}}\left[{Y}_{i,g}|{T}_{i},{N}_{\sigma ,i}\right]=\sum_{i:s\left(i\right)=s}\left[{\mu }_{{T}_{i},g}+{\mu }_{{T}_{i},g}\sum_{{\text{n}}=1}^{T}{N}_{\sigma ,i,n}{\beta }_{\sigma ,{t}_{i},n}^{g}\right]$$

Because all single cells in the same spot have the same effective niche and the sum only depends on the cell type of each cell, by summing over the niche cell type rather than each single cell we get an alternative sum of

$$\sum_{t}\left[{n}_{s,t}{\mu }_{t,g}+{n}_{s,t}{\mu }_{{T}_{i},g}\sum_{n=1}^{T}{N}_{\sigma ,i,n}{\beta }_{\sigma ,{t}_{i},n}^{g}\right]$$

Let ${\mu }_{s,g}=\sum_{t}{n}_{s,t}{\mu }_{t,g}$ and $\frac{{n}_{s,t}{\mu }_{t,g}}{{\mu }_{s,g}}={p}_{s,t,g}$. The sum above can be simplified to

$${\mu }_{s,g}+{\upmu }_{{\text{s}},{\text{g}}}\sum_{t}{p}_{s,t,g}\sum_{{\text{n}}=1}^{T}{N}_{\sigma ,i,n}{\beta }_{\sigma ,{t}_{i},{\text{n}}}^{g}$$

Moving terms in the sum we get that

$$\begin{array}{cc}& {\mathbb{E}}\left[{X}_{s,g}|{n}_{s,t},{N}_{\sigma ,s}\right]={\mu }_{s,g}+{\mu }_{s,g}\sum\limits_{t}\sum\limits_{n=1}^{T}{p}_{s,t,g}{N}_{\sigma ,s,n}{\beta }_{\sigma ,t,n}^{g}\end{array}$$

Subtracting ${\mu }_{s,g}$ and then dividing by ${\mu }_{s,g}$ on both sides of above, we have

$$\frac{{\mathbb{E}}\left[{X}_{s,g}|{n}_{s,t},{N}_{\sigma ,s}\right]-{\mu }_{s,g}}{{\mu }_{s,g}}=\sum_{t}\sum_{n=1}^{T}{p}_{s,t,g}{N}_{\sigma ,s,n}{\beta }_{\sigma ,t,n}^{g}$$

We expect the left-hand-side of the above to be small, and thus, using the same $x={\text{log}}(1+x)$ approximation, we get our final spot-level model for expected expression of gene $g$ in each spot conditioned on the spot’s cell-type composition and effective niche,

$${\text{log}}\left({\mathbb{E}}\left[{X}_{s,g}|{n}_{s,t},{N}_{\sigma ,s}\right]\right)={\text{log}}\left({\mu }_{s,g}\right)+\sum\limits_{t}\sum\limits_{t\mathrm{^\prime}=1}^{T}{p}_{s,t,g}{N}_{\sigma ,s,t\mathrm{^\prime}}{\beta }_{\sigma ,t,t\mathrm{^\prime}}^{g}$$

Note that, since ${\beta }_{\sigma ,t,{t}^\prime}^{g}$ is the same parameter carried through from model (1), it has the same interpretation as model (1), that is, it represents the effect of unit niche-composition increase of cell type ${t}^\prime$ on the single-cell expression of gene $g$ in cell type $t$.

### Parameter estimation in the Niche-DE model

#### Data pre-processing

To remove outliers in gene expression, for each gene, we cap gene expression to the 99.5 percentile across all spots.

####  Deconvolution and estimation of$n_{s,t}$

All samples used were deconvolved with RCTD using the “Multi” setting with maximum number of cell types in each spot equal to 4. We thresholded each deconvolution estimate to have a minimum value of 0.05. Because we found that deconvolution via RCTD overestimated the amount of tumor cells and hepatocytes in each spot, we thresholded each deconvolution estimate such that tumor cell and hepatocyte composition had a minimum value of 0.25. Spots that did not meet this criterion had tumor cell and hepatocyte composition set to 0.

Critical to the effective niche calculation is an estimate of the number of cells in each spot ${n}_{s,t}$. Assume that there are ${N}_{s}$ cells in spot $s$, for which the cell-type composition is ${\pi }_{s,t}$. From the reference dataset, let ${L}_{t}$ be the average library size of cell type $t$. The expected library size for spot $s$ is given by ${N}_{s}\sum_{t}{\pi }_{s,t}{L}_{t}$. Let ${L}_{s}$ be the observed library size of spot $s$. A natural estimate for ${N}_{s}$ given ${\widehat{\pi }}_{s,t}$, the estimate of the cell-type composition of spot $s$, is thus ${\widehat{N}}_{{\text{s}}}=\frac{{L}_{s}}{\sum_{t}{\pi }_{s,t}{L}_{t}}$. Using this estimate, we can then approximate ${n}_{s,t}$ by ${\widehat{N}}_{s}{\widehat{\pi }}_{s,t}$.

#### Joint analysis of multiple spatial transcriptomic data sets

Because niche-DE only depends on the effective niche, joint analysis of multiple spatial transcriptomic data sets, possibly of different resolutions, can be accomplished easily by ensuring that the effective niche is on the same scale across all data sets. We will demonstrate how to do this for two data sets, the first we call the reference dataset and the second we call the query dataset. The first step is to scale the coordinates of the query dataset such that one pixel in the query dataset corresponds to same physical distance as one pixel in the reference dataset. This makes sure the kernel bandwidth used has the same interpretation across both datasets.

If both datasets are of spot-level resolution, as is the case with 2 Visium datasets, we must scale the effective niche of the query dataset as well. The reason is that the estimated number of cells in each spot is determined by the library size of the spot. Since the niche-DE model is a regression on the effective niche, the effective niches of the query and reference dataset must be corrected to account for sequencing depth. To accomplish this, let the spot radius of the reference and query datasets be $r$ and $q$ respectively. Let ${N}_{r}$ and ${N}_{q}$ be the average number of cells in each spot of the reference and query datasets. We propose scale the effective niche of the query dataset by $({N}_{r}{q}^{2})/({N}_{q}{r}^{2})$. Scaling by ${N}_{r}/{N}_{q}$ corrects for differences in sequencing depth between the reference and query dataset. Scaling by ${q}^{2}/{r}^{2}$ makes sure that our correction is not driven by differences in spot sizes in the two datasets. For example, if a spot doubles in size, it will occupy 4 times the area and thus its library size is expected to be 4 times higher.

To address batch effects induced by data integration, we add batch indicators corresponding to each sample to our regression for each gene. This allows each gene to have a batch specific intercept term.

#### Normalization and filtering

In single-cell data, cells of the same type can have different library sizes. As such we must adjust ${\mu }_{i,g}$, the expected expression of gene g for single cell $i$, to account for this before applying niche-DE. Let ${L}_{{T}_{i}}$ be the expected library size of a cell of type ${T}_{i}$. Let ${L}_{i}$ be the library size of single cell $i$. We scale ${\mu }_{i,g}$ to be such that ${\mu }_{i,g}= {A}_{i,g}{L}_{i}/{L}_{{T}_{i}}$ w, where ${A}_{i,g}$ is the average expression of gene $g$ in cell type ${T}_{i}$ as given by the reference. This ensures that niche genes are not driven by patterns in sequencing depth. Note that in spot-level data, this scaling is a byproduct of the estimation of ${N}_{s}$ and its role in calculating ${\mu }_{s,g}$ the expected expression of gene g for spot $s$.

Let $N$ be the effective niche matrix of our dataset where $S$ is the number of spots and $T$ is the number of cell types. Before running the niche-DE model, we scale $N$ column wise so that each column has mean 0 and standard deviation 1. This alleviates biases in overall tissue cell-type composition so that the $\beta$ coefficients are on the same scale. We then center the columns to have mean 0. We also set ${p}_{s,t,g}=0$ if it is less than $0.05$. This was shown to increase numerical stability.

Also for the sake of numerical stability, we set ${\beta }_{i,n}^{g}=0$ unless three conditions hold. The first is that the total expression of gene $g$ across all spots is greater than some threshold $C$. The second is that there exists more than $M$ spots that contain index cell type $i$ which have an effective niche that contains niche cell type $n$. This is to ensure that signals found are not dominated by very few points. The third condition is that gene $g$ is in the top $\gamma$ percentile of expressed genes in cell type $i$. This is because most genes should have essentially 0 expression in a given cell type $i$. Default values for $M$, and $\gamma$ are 30 and 80 respectively. $C$ is dependent on the number of spots in the dataset but niche-DE is quite robust to the choice of $C$.

Finally, for computational efficiency, rather than using standard negative binomial regression which is quite slow, we instead perform standard Poisson regression using the “glm” package in R. Then with the given regression coefficients, we estimate the overdispersion parameter with the “optim” function in R. This was shown to work well as seen in Fig. 2A.

#### Parameter choice

Recall that the niche-DE has three parameters: $C$, the minimum expression across all spots necessary for a gene to be included in the regression, $M$, the minimum number of spots that contain the index cell type and have the niche cell type in its effective niche for an index-niche pair to be included in the regression, and $\gamma$, the minimum percentile a gene’s expression needs to attain in the index cell type to be included in the regression. For the integrated 10X VISIUM colorectal cancer data, we set $\left(C,M,\gamma \right)=\left(400,10,80\%\right)$. For the SMI NSCLC data, we set $\left(C,M,\gamma \right)=\left(6000,50,60\%\right)$. For the Slide-seq cerebellum data, we set $\left(C,M,\gamma \right)=\left(30,30,80\%\right)$. For the integrated 10X VISIUM kidney data, we set $\left(C,M,\gamma \right)=\left(300,20,80\%\right)$.

Niche-DE on the COSMX data was done with kernel bandwidths of 150, 250, 350, and 450 pixels which correspond to 30, 50, 70, and 90 µm respectively. Niche-DE on the slide-seq data was done with kernel bandwidths equal to the first, fifth, and tenth percentile of the total distance matrix of our dataset. Niche-DE on the integrated data was done with kernel bandwidths 1,100, and 250 which corresponded to bandwidths of 0.2, 100, and 250 µm.

### Downstream analysis

#### Reactome analysis

Let ${G}_{i,n}$ be the set of genes that are found to be ${\left(i,n\right)}^{+}$ niche genes. To infer what processes are active in index cell type $i$ in the presence of niche cell type$n$, we use “enrichR” [51] to perform a pathway analysis using ${G}_{i,n}$ as input. The database we use is “Reactome 2016”.

#### Marker gene analysis

Given index cell type $i$ and niche cell types ${n}_{1},{n}_{2}$, we define a gene $g$ to be a $\left(i,{n}_{1}\right)$ marker if the degree of upregulation in cell type $i$ in the presence of cell type ${n}_{1}$ is significantly greater than the degree of niche upregulation in the presence of cell type ${n}_{2}$. Statistically, this is equivalent to performing a contrast test with null hypothesis ${\beta }_{i,{n}_{1}}^{g}-{\beta }_{i,{n}_{2}}^{g}\le 0$ against alternative hypothesis ${\beta }_{i,{n}_{1}}^{g}-{\beta }_{i,{n}_{2}}^{g}>0$. Because niche-DE is based on a negative binomial regression, there is a closed form expression for the joint distribution of $\beta$. The distribution of ${{\widehat{\beta }}^{g}}_{i,{n}_{1}}- {{\widehat{\beta }}^{g}}_{i,{n}_{2}}$ can be shown to be normally distributed with a mean of ${\beta }_{i,{n}_{1}}^{g}-{\beta }_{i,{n}_{2}}^{g}$ and a variance that can be computed. We perform marker gene analysis to find marker genes in macrophages near tumor and hepatocytes on an integrated dataset containing 4 liver metastasized colorectal carcinoma Visium datasets.

#### Module score computation, labelling, and denoising of ScRNA-seq data

To evaluate the validity of our niche-DE tumor-associated macrophage marker gene set, we calculate the module score of this set of genes on liver CRC scRNA-seq data from Wu et al. [40] using the “AddModuleScore” function in Seurat applied to SAVER [35] denoised scRNA-seq values. Because the scRNA-seq data is not labelled, we perform label transfer using Seurat with the cells from the liver cell atlas [52] as the reference. Cells that were classified to be myeloid were extracted and label transfer was performed again using myeloid subtypes from the liver cell atlas as reference.

#### Ligand-receptor analysis

To determine which extracellular signaling mechanisms are driving niche-DE patterns between index cell type $i$ and niche cell type $n$, we developed a procedure integrating Niche-net [27] and Niche-DE statistics. The two statistics reflect different types of evidence supporting intra-cellular signaling: Niche-net provides a ligand-target matrix $A=\{{A}_{l,g}:l=1,\dots ,L;g=1,\dots ,G\}$, where $L$ is a set of ligands and $G$ is a set of target genes. ${A}_{l,g}$ reflects the confidence that ligand $l$ can regulate the downstream expression of gene $g$. For index cell type $i$, niche cell type $n$, and kernel bandwidth $\sigma$, Niche-DE provides a $G$ dimensional vector of one-sided *t*-statistics ${B}_{\sigma ,i,n}=\{{B}_{\sigma ,i,n,g}:g=1,\dots ,G\}$, where ${B}_{\sigma ,i,n,g}$ reflects whether or not gene $g$ is an ${\left(i,n\right)}^{+}$ niche gene operating at kernel bandwidth $\sigma$.

Studies suggest that different ligands tend to be effective over different distances [53]. To infer if cells of type $n$ within the niche of index cells of type $i$ are signaling to the index cells via ligand $l$, we first compute the optimal kernel bandwidth ${\sigma }_{l}^{*}$ for ligand $l$ based on the niche-DE regression likelihood score. This represents the most likely bandwidth at which we expect the ligand to operate and, by extension, the bandwidth at which we expect to observe downstream niche-DE genes. We then extract the top $K$ downstream target genes of ligand $\mathcal{l}$ from the Niche-net ligand-target matrix, which we call ${g}_{1},\dots {g}_{K}$. Using the Niche-Net ligand-target matrix values, we compute a weight vector ${W}^{\left(l\right)}$, where ${W}_{j}^{\left(l\right)}=\hspace{0.25em}{A}_{\mathcal{l},{g}_{j}}{\left(\sum_{k=1}^{K}{A}_{\mathcal{l},{g}_{k}}\right)}^{-1}$. Then, we combine these with the Niche-DE statistics to compute a ligand activity score ${T}_{\mathcal{l},i,n}^{U}=\hspace{0.25em}\sum_{j=1}^{K}{{W}_{j}^{\left(l\right)}B}_{{\sigma }_{l}^{*},i,n,{g}_{j}}$. If the top downstream genes $\{{g}_{1},\dots ,{g}_{K}\}$ are not niche-DE in index-niche configuration $\left(i,n\right)$, then ${T}_{\mathcal{l},i,n}^{U}$ should abide by the null distribution and thus be approximately normally distributed with mean 0 and variance $\sum_{j=1}^{K}{W}_{j}^{2}$. As such, we compute the standardize ligand activity scores, standardizing ${T}_{\mathcal{l},i,n}^{U}$ by its standard error under the null,

$${T}_{\mathcal{l},i,n}=\hspace{0.25em}\frac{{T}_{\mathcal{l},i,n}^{U}}{\sqrt{\sum_{j=1}^{K}{{W}^{\left(l\right)}}_{j}^{2}}}.$$

Thus, between index cell type $i$ and niche cell type $n$, we compute ${T}_{l,i,n}$ across all ligands $l$. We sort them in decreasing order, and let ${T}_{\left(M\right),i,n}$ be the ${M}^{\text{th}}$ largest order statistic amongst all ligand activity scores between index cell type $i$ and niche cell type $n$. We define the candidate ligand set as ${C}_{i,n}=\left\{\mathcal{l}:{T}_{\mathcal{l},i,n}>{\text{max}}\left(1.64,{T}_{\left(M\right),i,n}\right)\right\}$. The candidate ligand set represents the ligands with top evidence for being involved in signaling between index cell type $i$ and niche cell type $n$, based on the Niche-Net matrix and the niche-DE gene expression patterns between the two cell types.

Ligands in the candidate ligand set ${C}_{i,n}$ should be expressed by the niche cell type $n$, but this obvious condition is not a pre-requisite for its selection and still needs to be checked. This check is especially important since ligands may share similar downstream target genes, and thus, a ligand may have spurious high activity scores due to lack of specificity in its Niche-net profile. Therefore, we filter ligands and their receptors to ensure that the ligands are indeed expressed in the niche cell type $n$ and that the receptors are indeed expressed in the index cell type $i$, for $(i,n)$ pairs where ligand $l\in {C}_{i,n}$. This filter operates as follows: If a candidate ligand $\mathcal{l}$ is indeed expressed by cell type $n$ in the vicinity of index cell type $i$, there should be a positive correlation between the count of ligand $\mathcal{l}$ in a spot and the abundance of cell type $n$ in the spot, for spots that have index cell type $i$ in their effective niche. Thus, we perform a Poisson regression of the observed ligand expression ${X}_{s,\mathcal{l}}$ on the inferred spot-by-cell-type composition matrix $({n}_{s,{\text{t}}})$, yielding coefficient vector $({\beta }_{t}^{\mathcal{l}})$ for the enrichment of ligand $l$ within each cell type $t$. In this regression, we filter to only include those spots which have index cell type $i$ in their effective niche evaluated at bandwidth ${\sigma }_{\mathcal{l}}^{\star }$. From the regression, we get a *p*-value for testing ${H}_{0}:\hspace{0.25em}{\beta }_{n}^{\mathcal{l}}=0$ versus ${H}_{A}:{\beta }_{n}^{l}>0$, which we call ${p}_{n}^{\mathcal{l}}$. After applying the BH procedure over all candidate ligand *p-*values ${p}_{n}^{\mathcal{l}}$ for ligands in ${C}_{i,n}$ at a pre-set false discovery rate $\alpha$, we conclude that those with significant adjusted *p*-values are indeed expressed in niche cell type $n$ around index cell type $i$, and is reported as the set of “confirmed ligands” ${C}_{i,n}^{*}$. If the spatial data were single-cell resolution, we instead determine whether ligand $\mathcal{l}$ is in the top $\alpha$% of genes expressed by niche cell type $n$ that have cell type $i$ in their effective niche evaluated at bandwidth ${\sigma }_{\mathcal{l}}^{\star }$, with $\alpha$ specified by the user.

Using the ligand receptor list from Ramilowski et al. [54], we can furthermore curate a list of confirmed receptors for each ligand. Similar to above, to determine whether index cell $i$ expresses the corresponding receptor ${r}_{l}$ to confirmed ligand $\mathcal{l}\in {C}_{i,n}^{*},$ we perform a Poisson regression of the observed receptor expression ${X}_{s,{r}_{\mathcal{l}}}$ on the inferred spot composition matrix $({n}_{s,t})$ yielding coefficient vector ${\beta }_{t}^{{r}_{\mathcal{l}}}$. For this regression, we only include those spots which have niche cell type $n$ in their effective niche evaluated at bandwidth ${\sigma }_{\mathcal{l}}^{\star }$. This is to ensure that we only consider spots that can potentially receive ligand $\mathcal{l}$ emitted by niche cell type $n$. After computing *p*-values for ${\beta }_{t}^{{r}_{\mathcal{l}}}$ and performing the BH procedure over all known receptors of ligands in ${C}_{i,n}^{*}$, we conclude that those receptors with significant adjusted *p*-values are indeed expressed in index cell type $i$ in the vicinity of niche cell type $n$. If the spatial data were single-cell resolution, we instead determine whether receptor ${r}_{\mathcal{l}}$ is in the top $\alpha$% of genes expressed by index cell type $i$ that have cell type $n$ in their effective niche evaluated at bandwidth ${\sigma }_{\mathcal{l}}^{\star }$. If ligand $\mathcal{l}$ and its corresponding receptor ${r}_{\mathcal{l}}$ are confirmed to be expressed in the niche and index cell types, respectively, we then conclude that the ligand receptor pair $\left(\mathcal{l},{r}_{\mathcal{l}}\right)$ is active in signaling between index cell type $i$ and niche cell type $n$.

The user tunable parameters of this process are $K,$ the number of downstream genes to consider from the Niche-Net matrix, $M$, the number of candidates to include in the candidate ligand set, and $\alpha$, which is either the false discovery rate cutoff (for spot-level spatial data) or the expression rank cutoff (for single-cell resolution spatial data). We performed ligand-receptor analysis on the CosMx SMI NSCLC data as well as the integrated set of 10X Visium colorectal cancer datasets. We use $\left(K,M\right)=\left(50,50, 0.05\right)$ for the SMI data and $\left(K,M\right)=\left(25,50, 0.5\right)$ for the Visium data.

### Simulation designs

#### Bleeding simulations

##### Generic model

First, we describe how to simulate from the null model of no niche effects, where the gene expression of a spot/cell depends only on its cell-type composition/identity. Given a ST dataset, let ${n}_{s,t}$ be the estimated cell-type composition vector for spot $s$, i.e., ${n}_{s,t}$ is the proportion of cells at spot $s$ that are of cell type $t$. ${n}_{s,t}$ can be found via deconvolution [38]. If the data were single-cell resolution, then ${n}_{s,t}$ is simply a cell-type label, ${n}_{s,t}=1$ if cell $s$ is assigned label $t$, and ${n}_{s,t}=0$ otherwise. We multiply ${n}_{s,t}$ with the reference cell-type mean gene expression matrix to give us the expected expression vector ${\mu }_{s}=({\mu }_{s1}, \dots ,{\mu }_{sG})$, where ${\mu }_{sg}$ is the expected expression of gene $g$ at spot/cell $s$ without any niche effects. After library size normalization of ${\mu }_{s}$, we can sample an expression vector ${X}_{s}$ by drawing expression values based on a negative binomial distribution with mean ${\mu }_{s}$ and gene-specific overdispersion vector ${\gamma }_{g}$. The coordinates of simulated spot $s$ will be the same as the real coordinates of spot $s$. This can be done with any spatial transcriptomics data, but for our simulations, we use Visium from patient 4 (referenced below) and set ${\gamma }_{g}=1$ for all genes $g$.

##### Generic model incorporating niche effects

Let the assumed underlying spatial kernel bandwidth for gene $g$ be $\sigma$. Let ${N}_{\sigma ,s}$ be the effective niche vector for spot $s$ after appropriate normalization. Letting ${\beta }_{i,n}^{g}=\beta$ and ${\beta }_{i\mathrm{^{\prime}},n\mathrm{^{\prime}}}^{g}=0$ for all $i\mathrm{^\prime}\ne i$ and $n\mathrm{^\prime}\ne n$, we have that

$${\mu }_{s,g}^{\star }={\mu }_{s,g}{\text{exp}}\left({p}_{s,i,g}{\beta }_{i,n}^{g}{N}_{\sigma ,s,n}\right).$$

Thus, we sample ${X}_{s,g}$ by drawing expression values based on a negative binomial distribution with mean ${\mu }_{s,g}^{\star }$ and gene-specific overdispersion vector ${\gamma }_{g}$. To simulate datasets of size $B$, where $B$ is larger than the total number of cells/spots in the initial real data, we bootstrap $B$ samples from the set of $\left({N}_{\sigma ,s},{\mu }_{s}^{\star }\right)$. For each spot $s^\prime$ that is sampled, we simulate a spot with an expression vector ${X}_{s^\prime}^{B}$ sampled from a negative binomial regression with mean ${\mu }_{s{\prime},g}^{\star }$ and an effective niche equal to ${N}_{\sigma ,s^\prime}$. To test the power of niche-DE across varying effect sizes and sample sizes, we vary ${\beta }_{i,n}^{g}$ within $\{0.1,0.2,\dots ,1\}$ and vary $B$ within $\{1000,5000,10000\}$.

For our simulations, we use patient 4 (referenced below). We set $i$ to be fibroblasts, $n$ to be tumor, and $\sigma$ to be the first percentile of the total distance matrix of our dataset. We select 2000 random genes to be $\left(i,n\right)+$ niche genes.

##### Spatial bleeding model

Spot swapping is a known artifact in some spatial transcriptomic data sets where transcripts “bleed” into nearby spots, inducing artifactual correlation between the transcript counts in adjacent spots [7]. The severity of spot swapping varies across data sets. To examine how bleeding may affect niche-DE analysis, we add the bleeding effect to our simulation data according to SpotClean’s model [7]. In particular we let ${\alpha }_{g}$ be the gene specific local bleeding parameter. Let ${K}_{\tau }$ be a gaussian kernel with bandwidth $\tau$. Letting ${\mu }_{s,g}$ be the underlying expected expression of gene $g$ in spot $s$ with no bleeding effect and ${\mu }_{s,g}^{c}$ be the expected expression of gene $g$ in spot $s$ in the presence of bleeding, under the SpotClean model,

$${\mu }_{s,g}^{c}=\left(1-{\alpha }_{g}\right){\mu }_{s,g}+{\alpha }_{g}\sum_{s\mathrm{^\prime}}\frac{{K}_{\tau }\left(s,s\mathrm{^\prime}\right)}{\sum_{s^{\prime\prime} }{K}_{\tau }\left(s\mathrm{^{\prime}},s^{\prime\prime} \right)}{\mu }_{s\mathrm{^{\prime}},g}$$

We then sample the observed gene expression from a Negative Binomial with mean ${\mu }_{s,g}^{c}$ and gene-specific dispersion. We introduce this spatial bleeding effect to both the generic null model and the generic model with niche effects. This allows us to observe the effect of bleeding on both null *p*-values and the power of niche-DE. We set $\tau$ equal to the first percentile of the total distance matrix of our dataset.

Note that, with the spot-swapped simulation data, the procedure starts with a deconvolution which yields a contaminated estimate of spot cell-type composition ${n}_{s,t}^{c}$. This contaminated estimate ${n}_{s,t}^{c}$ is then used to generate a contaminated effective niche vector for each spot. Thus, the spot swap effect is “absorbed” in the cell-type composition estimation, which alleviates the influence on downstream niche-DE analysis.

##### Calculating runtime

Under the generic model with no niche effects, we perform niche-DE across three different kernel bandwidths in parallel across 4 cores on a laptop. We varied the number of cell types between {4,8,12}, the number of observations between {1000,2000,3000,4000,5000,10,000}, and the number of genes between {1000,5000,10,000}. To do this, we first took a 10X Visium dataset of metCRC with 4 cell types. To generate new cell types, we replicated the existing cell types to create cell subtypes that were identical to the 4 original cell types. Thus, each cell type either had 0, 1, or 2 replicates depending on the simulation. We then sampled either 1000, 5000, or 10,000 randomly from the genome, and simulated a new Visium dataset by sampling new cells at random to populate spots. The location of these spots was generated by sampling the coordinates from the original Visium dataset with replacement. Afterwards, each cell’s expression would be drawn from a negative binomial with the mean gene expression equal to the average expression found by a single-cell reference.

#### Pseudo-spot data generation and specificity and sensitivity analysis

##### Generating pseudo-spot data

To simulate lower-resolution datasets, we create pseudo spots by partitioning the field of view of a reference dataset into squares of side length $r$. All cells in the same square are assigned to the same spot. Gene expression in each spot is calculated by aggregating the gene expression of all cells in the spot. We create pseudo-spot data using the COSMX SMI NSCLC, Slide-seq cerebellum data, and Xenium breast cancer data as the initial datasets. The radii used for the COSMX data were 100, 125, 150, 175, 200, 225, and 250 pixels which corresponds to 20, 25, 30, 35, 40, 45, and 50 µm, respectively. The radii used for the slide-seq data were 10, 25, 30, 35, 40, 45, 50, 55, 60, 65, 70, and 75 pixels. The radii used for the Xenium data were 15, 20, 25, 30, 35, 40, 45, and 50 pixels.

##### Sensitivity and specificity calculations

Performing niche-DE on the initial high-resolution dataset, we obtain a set of significant $\left(i,n\right)$ niche genes at the gene, cell type, and interaction levels. Using this set of genes as a reference, we can perform niche-DE on the pseudo-spot dataset as well to calculate the sensitivity and specificity at all three levels.

Niche-DE on the COSMX SMI NSCLC pseudo-spot data was done with kernel bandwidths of 150, 250, 350, and 450 pixels which correspond to 30, 50, 70, and 90 µm respectively. Niche-DE on the slide-seq pseudo-spot data was done with kernel bandwidths equal to the first, fifth, and tenth percentile of the total distance matrix of our dataset. Niche-DE on the Xenium data was done with kernel bandwidths equal to the median distance between each cell and its nearest neighbor.

##### Ligand sensitivity calculations

To test the robustness of our ligand-receptor inference method, we apply the method to obtain the top $M$ ligands by activity score for the reference COSMX and slide-seq datasets as well as the corresponding pseudo-spot datasets. The reason we chose to compare ligand activity scores rather than candidate ligands or confirmed ligand-receptor pairs was because niche-DE applied to the lower-resolution datasets has less power than when it is applied to the original datasets. This makes the ligand potential scores lower which reduces the number of candidate ligands. As a result, the comparison of candidate ligand sets much noisier. By comparison, the set of top $M$ ligands is more robust as we still expect the ligand potential scores across resolutions to have a similar order. We choose $K$ = 50 and $M\in \{\mathrm{20,50}\}$.

### Datasets and data analysis

#### Published data sets

- *Slide-seq cerebellum*: We use the pre-deconvolved slide-seq cerebellum data [38]. This data can be found at https://singlecell.broadinstitute.org/single_cell/study/SCP948 and is the file named “myRCTD_cerebellum_slideseq.rds.” This dataset contained 19 cell types, 11,626 spots of spatial resolution 10 µm, and 5034 genes. For all analyses, we filtered the estimated cell-type composition vectors to only include the top 8 most ubiquitous cell types. This corresponded to astrocytes, Bergmann cells, fibroblasts, granule cells, MLI1, MLI2, oligodendrocytes, and Purkinje cells.
- *COSMX SMI non-small cell lung carcinoma*: We obtain Nanostring CosMx SMI non-small cell lung cancer data [29]. We used Lung sample 9 which contains two tissue sections measuring 960 genes with 87,684 and 139,735 cells and 22 cell types. Each sample spans a 5 mm by 4.25 mm area. Niche-DE and downstream analyses were performed on both samples in an integrative fashion. Permutation-based ligand-receptor analysis was done only on the section with 139,735 cells.
- *10X Visium (patient 4)*: This data contains liver metastasis of colorectal cancer. It has 848 spots and 36,601 genes.
- *10X Visium (patient 5)*: This data contains liver metastasis of colorectal cancer. It has 1663 spots and 36,601 genes.
- *CODEX (patient 4)*: CODEX data of liver metastasized colorectal cancer from patient 4 was obtained from Sathe et al. [39]. It contains 33,812 cells and 25 markers.
- *Wu *et al*. 10X Visium (Patients 1,2,3)*: We obtain 3 10X Visium data sets from liver metastasized colorectal cancer corresponding to patients 1, 2, and 4 of the study conducted by Wu et al. [40]. These datasets contain 3826, 4658, and 3721 spots with 36,601 genes. The dataset corresponding to patient 1 was found to have two tumor subclones found by Clonalscope.
- *Wu *et al*. scRNA-seq*: We obtain single-cell RNA-seq data from paired samples of colorectal cancer, adjacent colon, liver metastasis, and adjacent liver, lymph nodes along colons, and peripheral blood mononuclear cells (PBMC). After filtering the data to only include macrophage/monocytes as explained in “Niche-DE analysis identifies marker genes and signaling mechanisms specific to tumor-associated macrophages in liver metastasis” section, the final dataset contains 17,791 cells.
- *Colorectal cancer and liver metastasized colorectal cancer scRNA-seq*: For all 10X Visium colorectal cancer datasets, we merge scRNA-seq datasets from Sathe et al. [39]. The cell types present are hepatocytes and NK cells, cholangiocytes, T cells, B/plasma cells, tumor, endothelial, macrophages, and fibroblasts. Because all samples contained little to no lymphocytes and endothelial cells, we filter all deconvolution results to only include hepatocytes and cholangiocytes, tumor, macrophages, and fibroblasts.
- *Kidney Fibrosis 10X Visium*: We obtain 2 10X Visium data sets from fibrotic kidney. These datasets contain 2325 and 1375 cells with 36,601 genes. Deconvolution was performed using CellTrek.

#### Sample acquisition for Visium samples 4 and 5

This study was conducted in compliance with the Helsinki Declaration. Patients were enrolled according to a study protocol approved by the Stanford University School of Medicine Institutional Review Board (IRB-44036). Written informed consent was obtained from all patients. Samples were surgical tumor resections.

#### Tissue processing

Tissues were placed in cryomolds containing chilled TissueTek O.C.T. Compound (VWR). Additional O.C.T. was added to cover the tissue and cryomold was placed on powdered dry ice. Blocks were sealed and stored at −80 °C.

#### 10 × Visium Spatial transcriptomics library preparation

Libraries were prepared using Visium Spatial Gene Expression Reagent Kit (version 1) (10X Genomics). All steps were performed according to the manufacturer’s protocol. Briefly, 10-µm-thick tissue sections were placed onto a Visium Spatial Gene Expression slide using a cryostat. Following methanol fixation, hematoxylin and eosin staining was performed, coverslip was mounted, and slides were imaged using a Leica DMI 6000 or Keyence BZ-X microscope. A permeabilization time of 18 min was used, which was determined using the Visium Spatial Tissue Optimization Reagents Kit (version 1). Libraries were sequenced on Illumina sequencers (Illumina, San Diego, CA). Cell Ranger (10 × Genomics) version 5.0.0 “mkfastq” command was used to generate Fastq files. Space Ranger version 1.2.1 “count” was used with default parameters and alignment to GRCh38 to perform image alignment, tissue detection, barcode and UMI counting, and generation of feature-barcode matrix.

### Supplementary Information

**Additional file 1: Table S1.** Ligand-receptor pairs between CD8T cells and tumor cells found by niche-LR in the CosMx NSCLC data. **Table S2.** Ligand-receptor pairs between CD8T cells and tumor cells found via permutation test in the CosMx NSCLC data.

**Additional file 2: Table S1.** Description of data quality for liver metastasis 10X Visium data.

**Additional file 3: Figure S1.** Deconvolution plots for all 10X Visium liver metastasis samples. The heatmaps shown show the deconvolution results using RCTD when applied to liver patients 1, 2, 3, 4, and 5. The name of the cell type above each figure corresponds to the spot level deconvolution result for that cell type (i.e The Tumor headline shows the deconvolution results for tumor cells for each spot).

**Additional file 4: Table S1.** A list of (fibroblast, tumor)+ genes found by niche-DE in the integrated 10X liver metastasis colorectal cancer data. **Table S2.** The list of enriched pathways found by enrichR using the gene list in table S1. **Table S3.** The list of ligand-receptor pairs found by niche-LR for fibroblasts near tumor cells.

**Additional file 5: Table S1.** A list of (tumor, fibroblast)- genes found by applying niche-DE only in patient 4 of the manuscript. **Table S2.** The CODEX protein panel used to generate the CODEX data for patient 4 in the manuscript.

**Additional file 6: Figure S1.** A Venn diagram of genes found by niche-DE and genes found by C-side in the 10X Visium dataset belonging to patient 1. The venn diagram shown shows the total number of genes found to have niche effects when using Niche-DE vs C-Side on the liver patient 1 10X Visium data. We include separate diagrams for both (i,n)+ and (i,n)- genes.

**Additional file 7: Table S1.** A list of gene markers for fibroblasts when near tumor subclone 1 as opposed to tumor subclone 2 as found by niche-DE. **Table S2.** A list of gene markers for fibroblasts when near tumor subclone 2 as opposed to tumor subclone 1 as found by niche-DE. **Table S3.** The list of enriched pathways found by enrichR using the gene list in table S1. **Table S4.** The list of enriched pathways found by enrichR using the gene list in table S2.

**Additional file 8: Figure S1.** H&E annotation of 10X Visium liver metastasis patient 1. The image shown is a pathology annotation for the 10X visium patient 1 liver data. The spots in green correspond to spots that contain tumor cells.

**Additional file 9: Table S1.** A list of gene markers for macrophages when near tumor cells as opposed to hepatocytes as found by niche-DE. **Table S2.** A list of gene markers for macrophages when hepatocytes as opposed to tumor cells as found by niche-DE. **Table S3.** A list of ligand-receptor pairs found by niche-LR for macrophages when near tumor cells. **Table S4.** A list of (macrophage, tumor)+ genes found by niche-DE in the integrated 10X liver metastasis colorectal cancer data. **Table S5.** The list of enriched pathways found by enrichR using the gene list in table S4. **Table S6.** The list of enriched pathways found by enrichR using the gene list in table S1.

**Additional file 10: Table S1.** (Fibroblast, Proximal Tubular)+ cells as found by niche-DE in the integrated 10X Visium fibrotic kidney data. **Table S2.** Ligand-receptor pairs between fibroblasts and proximal tubular cells found by niche-LR in the integrated 10X Visium fibrotic kidney data. **Table S3.** The list of enriched pathways found by enrichR using the gene list in table S1. **Table S4.** (Proximal Tubular, Fibroblast)+ cells as found by niche-DE in the integrated 10X Visium fibrotic kidney data.

**Additional file 11.** Review history.

## Data Availability

*Software availability* The current source code for niche-DE can be found at http://kmason23.github.io/NicheDE/. The version of the source code used in the manuscript can be found at in Zenodo (DOI:10.5281/zenodo.10369260). Integrated Liver analysis: The code and data used to generate results and figures corresponding to the integrated liver analysis can be found on figshare (DOI:10.6084/m9.figshare.24803352). Integrated kidney analysis: The code and data used to generate results and figures corresponding to the integrated liver analysis can be found on figshare (DOI:10.6084/m9.figshare.24803166). Liver Subclone analysis: The code and data used to generate results and figures corresponding to the liver subclone analysis can be found on figshare (DOI:10.6084/m9.figshare.24803364). Liver patient 4 analysis: The code and data used to generate results and figures corresponding to the analysis of liver patient 4 can be found on figshare (DOI:10.6084/m9.figshare.24803325). *Data availability* Wu et al. 10X Visium Data (patients 1, 2, 3) and scrna-seq data: Per the manuscript in reference 40, the data can be found in the National Omics Data Encyclopedia at https://www.biosino.org/node/project/detail/OEP001756. Patient 4 10X Visium: The 10X Visium data for patient 4 is available on figshare (DOI:10.6084/m9.figshare.24803571). Patient 5 10X Visium: The 10X Visium data for patient 5 is available on figshare (DOI:10.6084/m9.figshare.24803616). Slide-seq Cerebellum: Slide-seq V2 data is available at the Broad Institute Single Cell Portal https://singlecell.broadinstitute.org/single_cell/study/SCP948. COSMX SMI Non-small Cell Lung Carcinoma: The CosMx NSCLC data can be found at http://nanostring.com/CosMx-dataset. Colorectal cancer and liver metastasized colorectal cancer scRNA-seq: The scrna-seq datasets used to create our reference dataset can be found at https://dna-discovery.stanford.edu/publicmaterial/datasets/mCRC_scRNAseq/mCRC_scRNA_filtered.zip. Kidney Fibrosis 10X Visium samples: The 10X Visium kidney data is available on GEO (accession number GSE211785).
