# Supplementary material for: Niche-DE: niche-differential gene expression analysis in spatial transcriptomics data identifies context-dependent cell-cell interactions
Source: Genome Biol. 2024 Jan 12;25:14. doi: 10.1186/s13059-023-03159-6 (PMC10785550; doi:10.1186/s13059-023-03159-6)

S1

Patient 1 Deconvolution

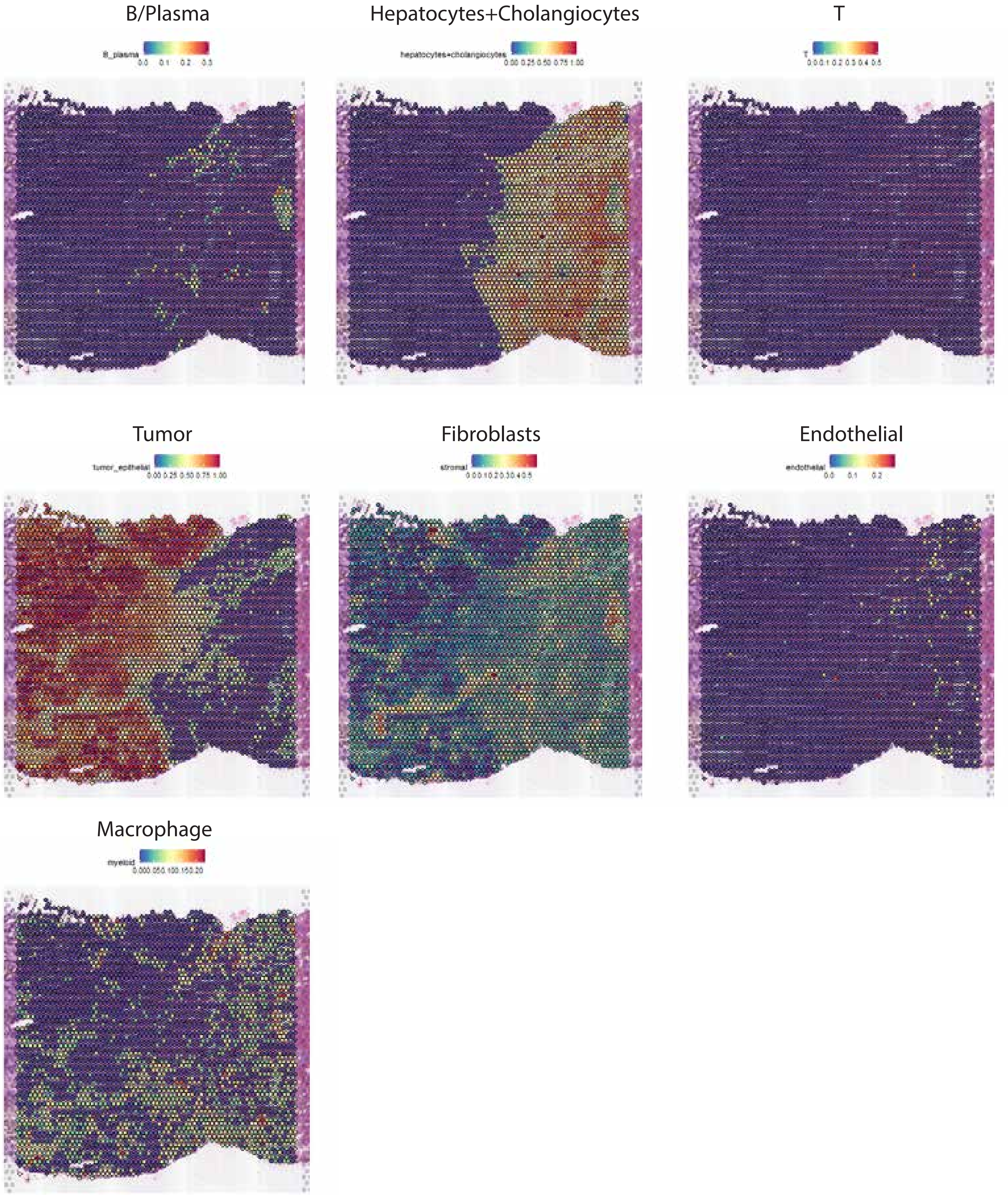

Patient 2 Deconvolution

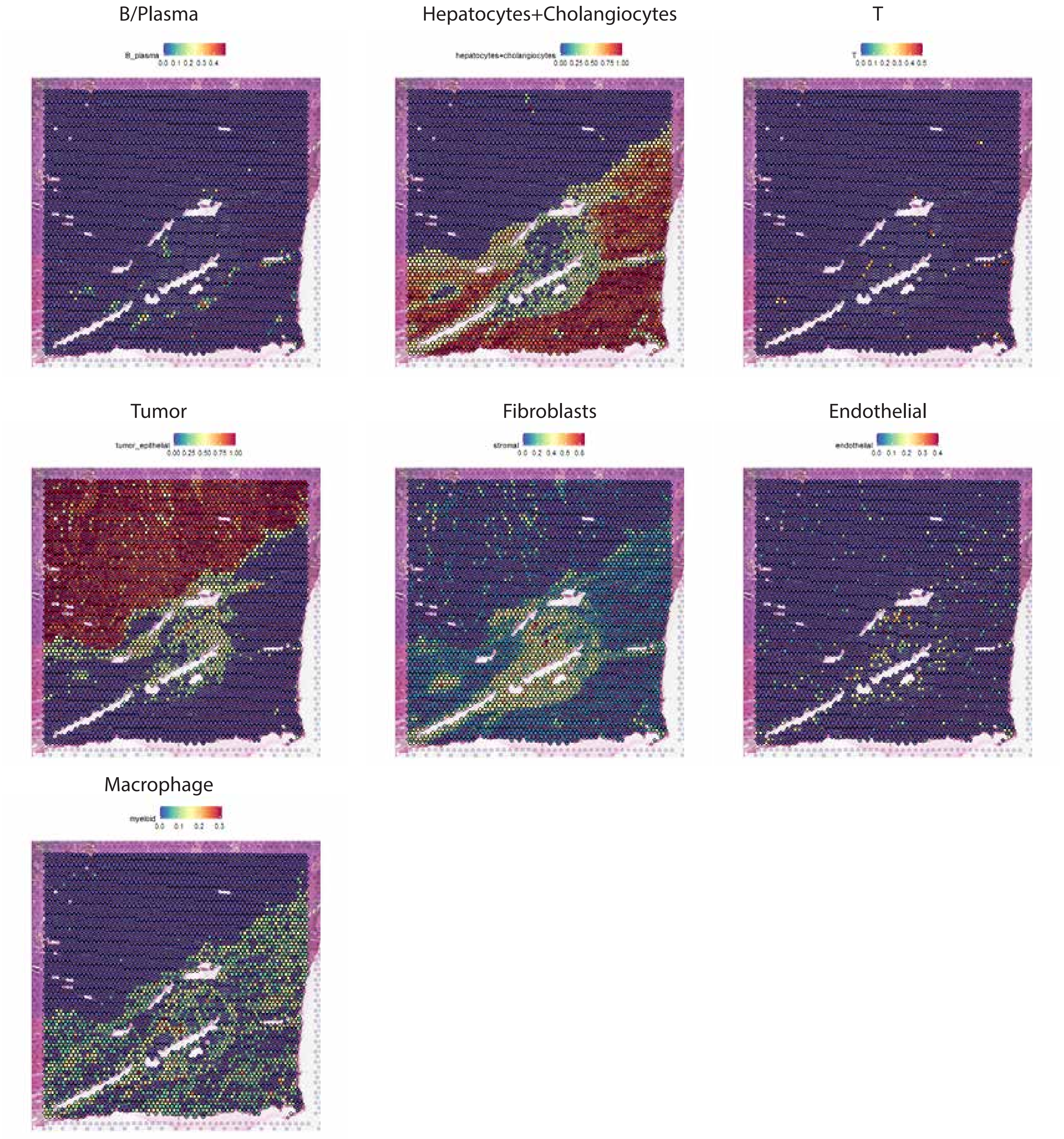

Patient 3 Deconvolution

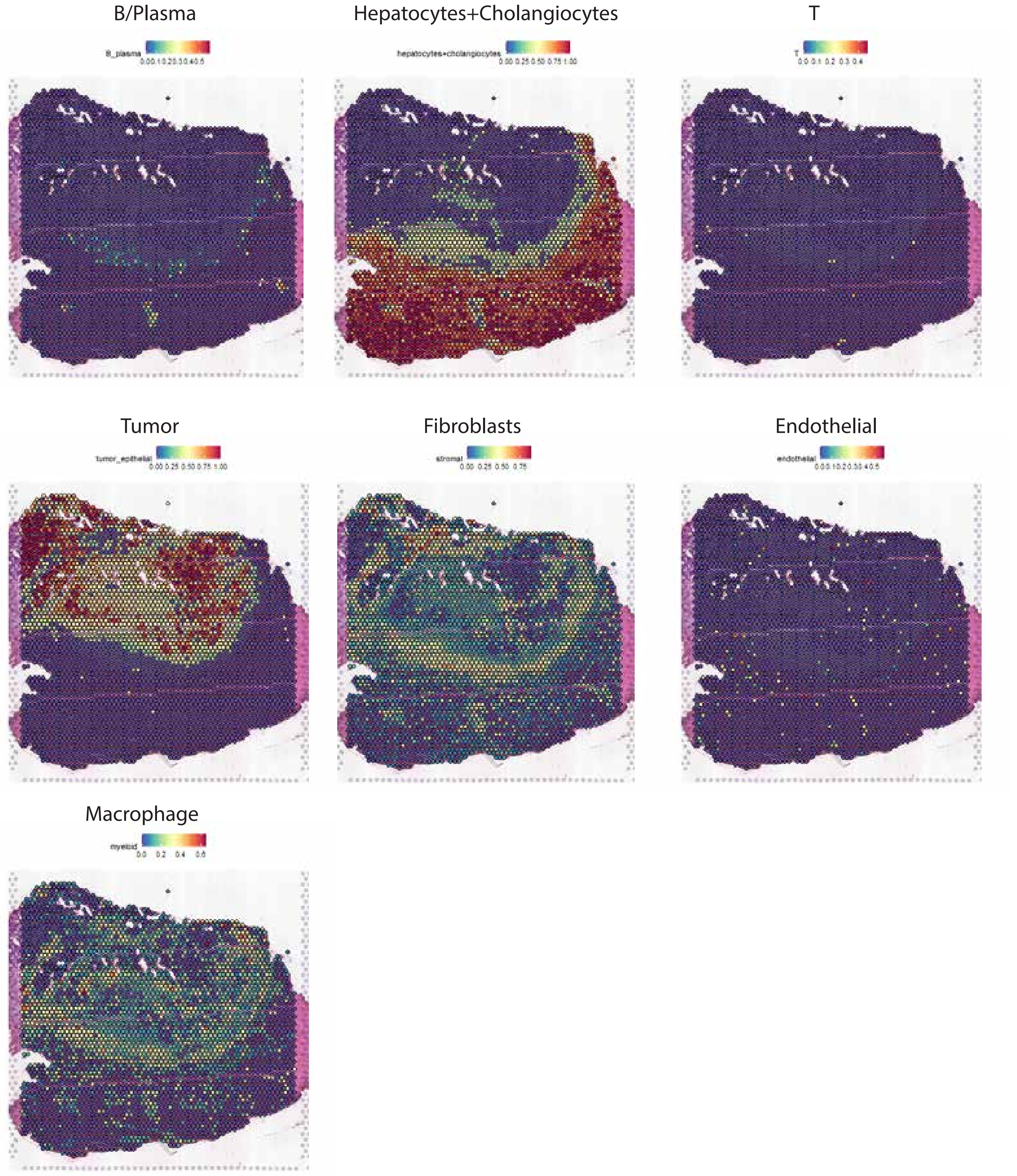

Patient 4 Deconvolution

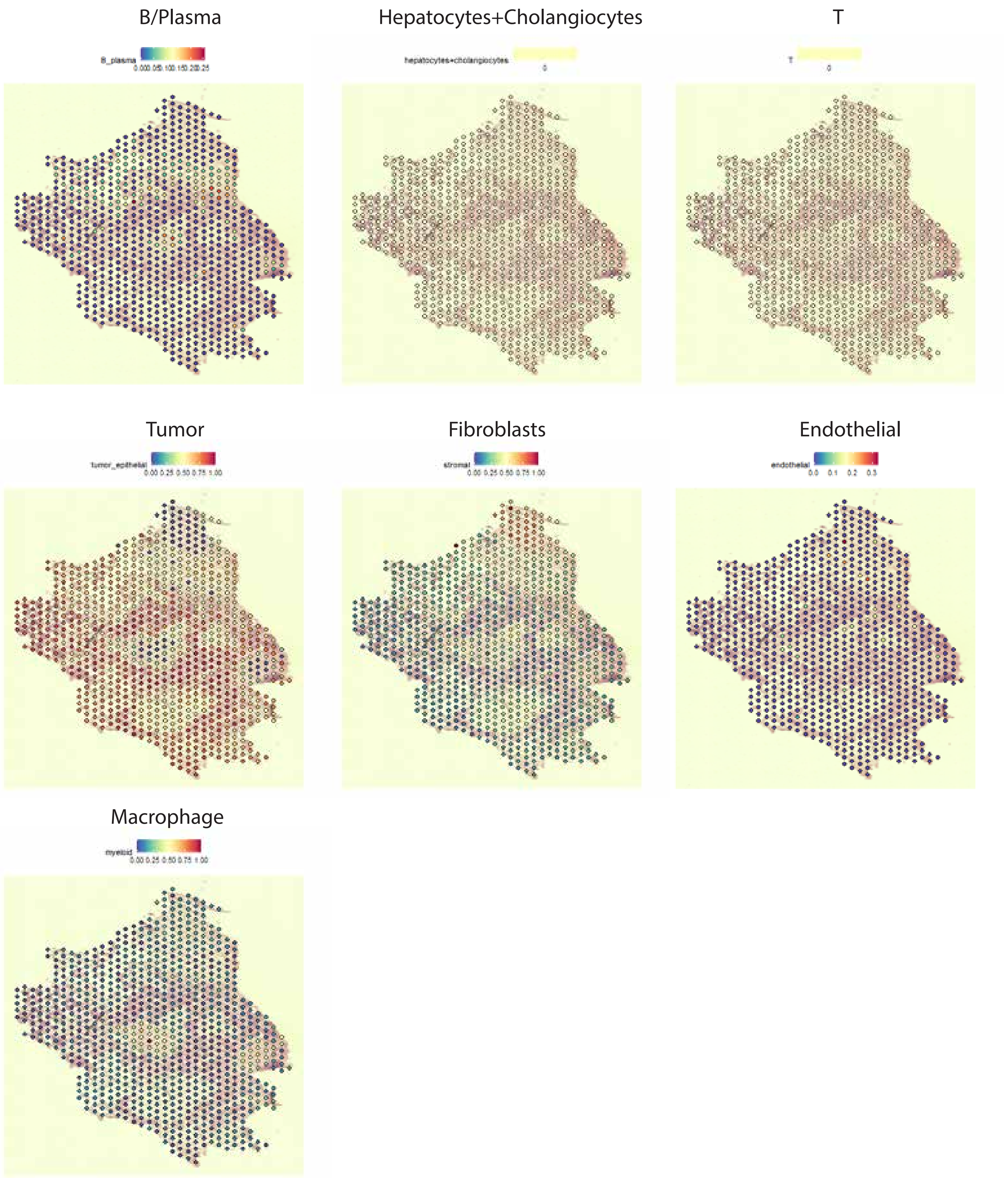

Patient 5 Deconvolution

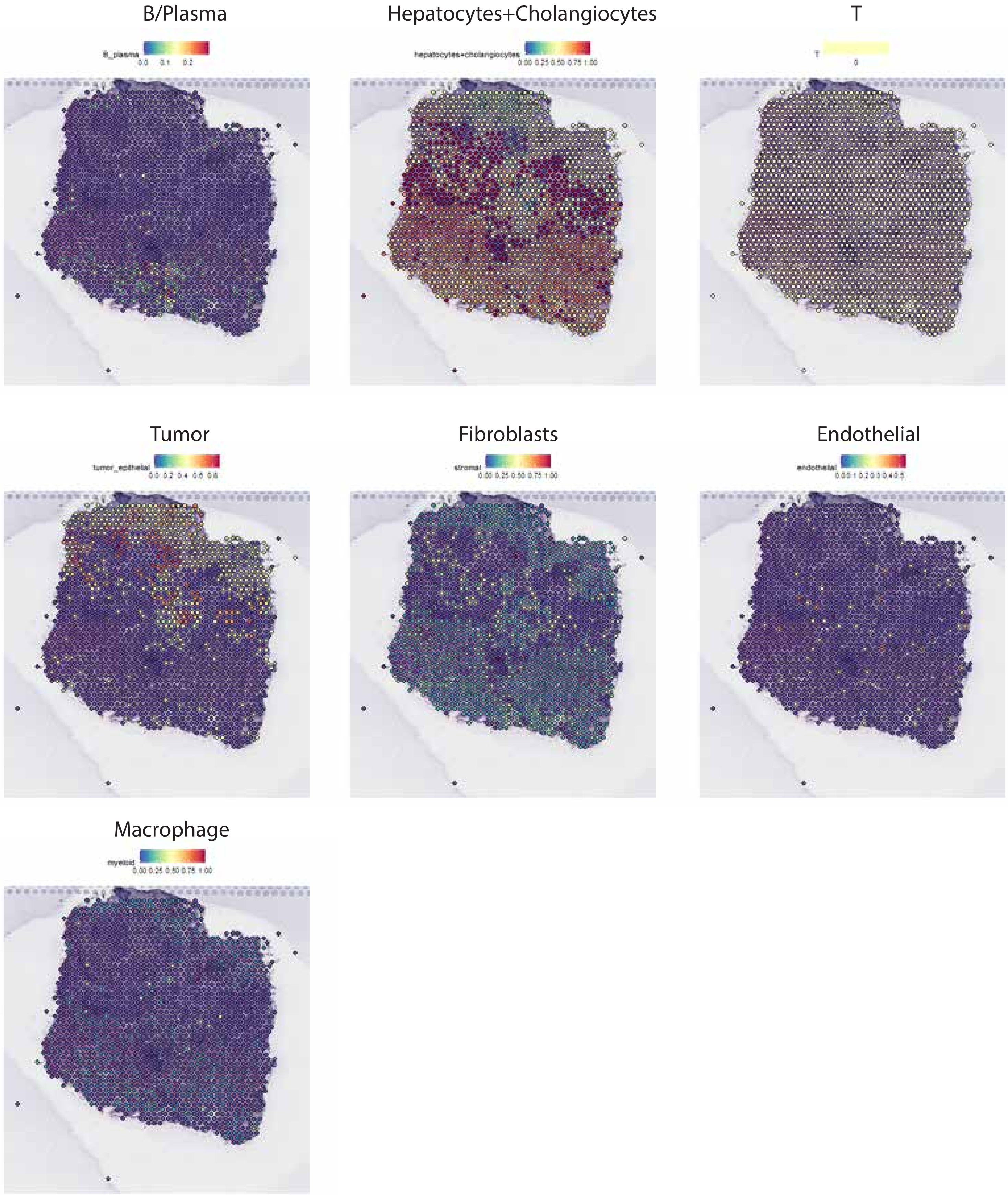

Supplement: Supplementary file 3 — Additional file 3: Figure S1. Deconvolution plots for all 10X Visium liver metastasis samples. The heatmaps shown show the deconvolution results using RCTD when applied to liver patients 1, 2, 3, 4, and 5. The name of the cell type above each figure corresponds to the spot level deconvolution result for that cell type (i.e The Tumor headline shows the deconvolution results for tumor cells for each spot). [file 13059_2023_3159_MOESM3_ESM.pdf]
