# Supplementary figures and images for: Niche-DE: niche-differential gene expression analysis in spatial transcriptomics data identifies context-dependent cell-cell interactions
Source: Genome Biol. 2024 Jan 12;25:14. doi: 10.1186/s13059-023-03159-6 (PMC10785550; doi:10.1186/s13059-023-03159-6)

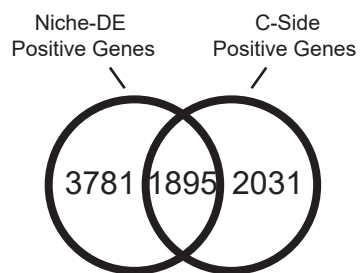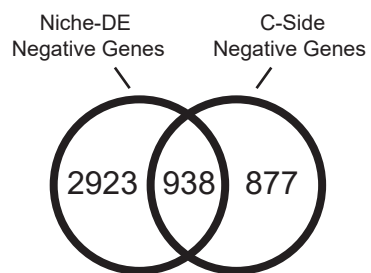

Supplement: Supplementary file 6 — Additional file 6: Figure S1. A Venn diagram of genes found by niche-DE and genes found by C-side in the 10X Visium dataset belonging to patient 1. The venn diagram shown shows the total number of genes found to have niche effects when using Niche-DE vs C-Side on the liver patient 1 10X Visium data. We include separate diagrams for both (i,n)+ and (i,n)- genes. [file 13059_2023_3159_MOESM6_ESM.pdf]
