# Supplementary material for: Niche-DE: niche-differential gene expression analysis in spatial transcriptomics data identifies context-dependent cell-cell interactions
Source: Genome Biol. 2024 Jan 12;25:14. doi: 10.1186/s13059-023-03159-6 (PMC10785550; doi:10.1186/s13059-023-03159-6)
Supplement: Supplementary file 11 — Additional file 11. Review history. [file 13059_2023_3159_MOESM11_ESM.docx]

**1^st^ round**

**Reviewer 1**

In this study, Mason et al. introduce a new method, niche-DE, for identifying cell type-specific genes with significantly up or down-regulated expression in the context of specific spatial niches. The niche-DE method has both single-cell-level and spot-level models. Building on Niche-DE, the authors also developed Niche-LR, a method to detect ligand-receptor signaling between cell types. They illustrated and benchmarked their new methods using multiple spatial datasets. While, the study presents a useful resource for the research community, there are some limitations. The method and manuscript can be improved by addressing the following comments/concerns:

Major comments:

• The authors applied niche-DE and niche-LR to the integrative analysis of five 10X Visium samples obtained from liver metastases of colorectal carcinoma. The liver metastases of colorectal cancer have relatively lower architectural complexity. In these samples, tumor regions are relatively easy to identify and less mixed with stromal tissues or immune cells, compared to primary tumors with complex tumor, immune and stromal cells. It is important to know the performance of niche-DE and niche-LR in primary tumors, such as primary pancreatic cancer, gastric cancer, or non-small cell lung cancer, which are rich in cancer-associated fibroblasts and macrophages.

• I would also suggest the authors benchmark niche-DE and niche-LR using the public Xenium and Merfish datasets and compared to the CosMx datasets. Such comparison will help determine how gene multiplexity and gene panel size influence the performance of niche-DE and niche-LR.

• There are likely other published methods on spatial gene expression and cell-cell interaction analysis. I suggest the authors compare niche-DE and niche-LR with existing methods to determine if their new methods outperform current approaches.

• The images in Fig. 4 and fig. 5 in the merged PDF file are low resolution and blurry, making it difficult to review the details. High-resolution H&E images for these tissue sections are not provided, and the pathologist annotations and color key are also missing.

• For spatial transcriptomic data at single-cell resolution, such as the CosMx data, it is unclear how the authors handled doublets and gene dropout issues, which are essential for both niche-DE and niche-LR analysis. A more detailed explanation of the approach taken to address these challenges would be helpful for readers.

• Regarding the index and niche cell type annotations, what is the level of granularity? Does it focus on major cell types (e.g., fibroblasts, T cells, B cells, macrophages), or can it go a big further to distinguish specific cell states (e.g., cell states such as M2-like macrophages, regulatory T cells, exhausted T cells, naïve T cells, etc.)? Understanding cell-cell communications between various cell states is more important compared to just focusing on major cell types.

• Regarding niche differential expression (niche-DE) analysis, it is important to understand how the spatial niches are defined. What is the relationship between spatial niches and annotated cell types? Is a spatial niche an aggregate of a certain type of cells (with 100% cell type purity within a spatial niche)? or is it an aggregate of cells with mixed cell types? In the real-world scenarios, many immune cell types do not always cluster together and form a pure niche; instead, they are often intermixed. A clear explanation of the spatial niche definition would be helpful for readers.

• When integrating data from multiple tissue slides and across patients, how does niche-DE account for tissue- and slide-batch effects? It is important to assess the performance of niche-DE in correcting tissue- and slide-batch effects.

• Compared to spatial domains defined based on spatially variable gene expression analysis, what are the main advantages of niche-differential expression analysis? the study does not provide a comparison with existing methods on spatial clustering analysis. clarifying the strengths of niche-DE over these methods would help emphasize its value.

• The manuscript claims that their methods can also be applied to ROI-based spatial transcriptomics data, but it does not provide any data on ROI-based spatial transcriptomics data.

• When applying niche-DE to lower resolution spot-level data, the authors deconvolved each sample using the single cell reference data set. If the spot-level ST data lack matched single-cell RNA-seq data, will this limit the application of niche-DE? Generating paired single-cell and spatial data for the same samples can be very challenging, due to high cost and insufficient tissue availability. In addition, most spatial data are generated using FFPE tissues, which cannot be used to generate single-cell RNA-sequencing data. If niche-DE can still be used to analyze the spot-level ST data without matched single-cell data, what deconvolution method can be employed? It would be helpful if the authors could benchmark their methods using existing datasets. additionally, how are unique gene signatures defined for deconvolution analysis? it is important to consider that some markers can be expressed by more than one cell types.

• The Discussion is largely restating their results.


Minor comments:

• Lines 23-24 and Line 32, the references cited are somewhat outdated, and recent significant publications have not been included.

• For the CosMx SMI NSCLC data used in this study, how many samples and tissue sections are used?

**Reviewer 2**

Summary

The authors present niche-DE, which is a new method for differential gene expression analysis in the context of local spatial niches consisting of mixtures of cell types in spatial transcriptomics data. This is an innovative method, which fills an important gap in the existing literature for spatial transcriptomics analysis methods, which largely focus on global analysis across whole tissue sections. The method is statistically sophisticated and rigorous, comprehensively described, and the authors evaluate it on both simulated and real datasets from both cell-level and spot-level platforms. Overall, this new method is an excellent addition to the literature on spatial transcriptomics analysis methods, which will be of high interest for readers. Several specific comments are described below, largely regarding the computational implementation and reproducibility of analyses, and some clarifications on the use cases of the method.


Major comments

* The analysis for spot-level data (e.g. 10x Genomics Visium) relies on first applying spot-level deconvolution to identify the cell types per spot. Could the authors provide some discussion of what would happen if the spot-level deconvolution does not perform well?

* Related to the previous point, it would also be useful to include some more discussion of the types of data that the method is applicable to. For example, the cancer and cerebellum examples in the text are characterized by a diversity of major cell types. Would the method work in datasets from other biological systems that contain more subtle cell type differences (e.g. subpopulations of neurons in the cortex)?

* The runtime / computational scalability of the method are not clear. It would be useful for readers to include some results demonstrating the runtime / computational scalability, and providing some details on the largest dataset sizes that can feasibly be analyzed (e.g. number of cells and genes), and the type of computational hardware required (laptop or high-performance compute cluster, number of cores).

* While the authors have set up an online vignette / tutorial to demonstrate usage of the method, the examples in this tutorial currently either return zero results (niche-DE genes and marker genes) or give errors (pathway enrichment and ligand-receptor analysis) when using the example dataset provided (raw data matrices). It would be useful to provide a tutorial that generates some meaningful results and/or plots, which users can then adapt for their own analyses.

* Code and data to reproduce the analyses in the manuscript are unavailable. These should be provided in publicly accessible repositories.


Minor comments

* A short description / definition of the term "niche" in the introduction would be helpful.

* Installation and loading of the package in R returns a warning message, which should be addressed.

* Some citations are missing in the last paragraph of the introduction (currently showing placeholders "[CITE]").

**Authors’ response**

Reviewer 1

Major comments:

• The authors applied niche-DE and niche-LR to the integrative analysis of five 10X Visium samples obtained from liver metastases of colorectal carcinoma. The liver metastases of colorectal cancer have relatively lower architectural complexity. In these samples, tumor regions are relatively easy to identify and less mixed with stromal tissues or immune cells, compared to primary tumors with complex tumor, immune and stromal cells. It is important to know the performance of niche-DE and niche-LR in primary tumors, such as primary pancreatic cancer, gastric cancer, or non-small cell lung cancer, which are rich in cancer-associated fibroblasts and macrophages.

Thanks for this suggestion, and we agree that it makes sense to demonstrate our method on more diverse data types.  It is a good idea to do a primary cancer scenario, but since the examples in the initial submission were all cancer-related, we thought it would be even better to do a case study in a non-cancer scenario.  Thus, we added an example from a study on chronic kidney disease (CKD). This dataset has many distinct spatial domains comprised of lymphocytes, fibroblasts, myeloid cells, and kidney-specific cells such as proximal tubular cells.  Since there is a lot of existing knowledge on CKD relating to the role of fibroblasts and proximal tubule cells, we used an analysis of these two cell types as a test of whether niche-DE can uncover existing knowledge.  The results on fibroblasts and proximal tubular cells are given in a new Figure 6, and niche-DE results between other cell types are in the supplementary.

• I would also suggest the authors benchmark niche-DE and niche-LR using the public Xenium and Merfish datasets and compared to the CosMx datasets. Such comparison will help determine how gene multiplexity and gene panel size influence the performance of niche-DE and niche-LR.

In the revision, we added a benchmark using Xenium alongside those using CosMx and Slide-Seq V2, in Figure 2F.  We want to note that, in order to assess power and specificity, we need to be in a scenario where we know which are the true niche-DE genes.  With current technology, this is only possible in simulations, and that is what we show in Figure 2B,C.  We also included an alternative validation using CODEX in Figure 4C,D, but that type of validation is only possible for the very few genes for which we have antibodies, and thus, does not allow a systematic assessment of power across the broad spectrum of signal types.  Thus, for the publicly available Xenium data, we benchmarked the performance of niche-DE by varying pseudo-spot resolutions. The results show that although the Xenium data is a different technology on a different tissue, it has similar trends as CosMx and Slide-seqV2.

• There are likely other published methods on spatial gene expression and cell-cell interaction analysis. I suggest the authors compare niche-DE and niche-LR with existing methods to determine if their new methods outperform current approaches.

Thanks for this question, as it leads to an important discussion:  Niche-DE, cell-cell interaction analysis, and spatially variable gene analysis are very different types of analyses on spatial transcriptomics data, aiming at different goals.  To clarify the differences between these methods, we added more details at how these analyses differ in the Discussion and Introduction of the revision.  Because of their differing goals, it is not clear whether and how these analyses methods should be benchmarked against each other.

Specifically, in the Introduction, we wrote:  “We would like to highlight the differences between niche differential expression analysis and common spatial analyses such as spatially variable gene analysis and cell-cell interaction analysis.  Spatially variable gene analysis finds genes that show expression patterns that correlate with some spatial pattern while cell-cell interaction analysis looks for cell types that colocalize with each other more than expected by random chance.  While the former does not explicitly consider the colocalization of cell types present in the tissue, the latter is a function of the cell types and their locations with no regards to gene expression.  In contrast, niche-differential expression analysis finds genes in an index cell type (e.g macrophages) that show expression patterns that correlate with its colocalization with another cell type (e.g tumor cells).  This gives insight to how colocalization of two cell types affects their gene expression state.   The method that is most related to niche-DE in terms of analysis goal is C-Side, but that also has substantial differences, which we will discuss more under Results and Discussion. ”

We also added the following section to the Discussion: “Currently, two of the most common analyses of spatial transcriptomic data are spatially variable gene analysis and celltype colocalization analysis.  In the Introduction, we expounded on the differences between these analyses.  We stress here that the conclusions made from a niche-DE analysis do not subsume that of a spatially variable gene analysis or a cell-cell interaction analysis. A gene that is an (i,n)^+ niche gene may not be a spatially variable gene if the index and the niche cell type do not colocalize according to some spatial pattern. Further, the index cell and niche cell do not need to colocalize more than random in order to have niche-DE genes between them.. Therefore, we recommend that niche-DE be used in conjunction with, not as an alternative to current spatial analyses.”

Among all of the published methods, the method that bears some similarity to niche-DE is C-side (Cable et al., Nature Methods 2022, reference #28 in paper). However, even these two methods are not directly comparable as niche-DE does much more than C-side.  In the Results and Discussion of the revision, we highlight the differences between the two methods:

In the Results: “... Among published methods, the method that is most similar to niche-DE in terms of analysis goal is C- Side28.  There are important methodological differences between niche-DE and C-side.  By starting from a single cell model and then using it to derive a spot-level model, niche-DE gives explicit interpretation of the parameter β_(i,n)^g, which is the increase in expression of gene g in index cell type  i per unit increase in the niche proportion of cell type n.  Although C-Side also aims to detect cell-type specific differentially expressed genes, its model does not admit to this explicit interpretation of parameters.  Niche-DE also accounts for the average library size of each cell type in performing differential expression analysis.  This is important because C-side assumes that the change in gene expression for a spot is equal to the average change across all cell types in the spot.  Niche-DE also differs from C-side in the way that it treats multiple cell types, the unknown spatial neighborhood size, and the massively parallel multiple tests, as we detail in the Discussion…”

“For comparison, we also applied C-Side to the Visium sample from patient 1.  Since C-Side requires the user to input the covariate matrix, and does not give instructions on what to use, we used as input the effective niche computed by niche-DE.  C-Side does not allow combining evidence across multiple neighborhood sizes, and thus we used a fixed kernel width of 250 pixels. Next, we extracted the niche-specific coefficients and standard errors from C-Side to manually compute their p-values and perform FDR control at the 0.05 level via Benjamini Hochberg procedures, giving us 3926 upregulated and 1815 downregulated interaction-level signals. Niche-DE gives 5676 upregulated and 3861 downregulated interaction-level signals. The venn diagram of discoveries made between the two methods can be found in Supplementary figure 8.1.  Even when the two methods both start with the covariate matrix computed by niche-DE, there are significant differences, which we believe is due partly to the differences discussed above (i.e. niche-DE adjusts for cell type-specific library size and conducts hierarchical FDR control, which is not performed in C-Side).

An inspection of (fibroblast,tumor)+ genes found by niche-DE and C-Side shows that C-Side finds 167 genes and niche-DE find 319 genes with 57 genes overlapping between both gene sets. Pathways associated with genes found by C-Side but not niche-DE correspond to metabolism whereas pathways associated with genes found by niche-DE but not C-Side correspond to translation, extracelluar matrix organization, collagen formation, and metabolism. 50 genes found by niche-DE but not C-Side code for ribosomal proteins, which should be upregulated in fibroblasts undergoing tumor-associated extracellular matrix remodelling.”

And finally, in the Discussion:  “Among existing methods, the method that is most similar to niche-DE in terms of analysis goal is C-Side28.  However, there are important differences, as highlighted under Results.  We want to note here that in addition to differences in the model, niche-DE allows for automatic consideration of multiple gene-specific kernel bandwidths and integrated hierarchical FDR control. Since we do not know the optimal bandwidth size a priori, we must test multiple bandwidths and combine the results to come up with a single pvalue. Analysis of multiple bandwidths and the corresponding pvalue combination step is automatic in niche-DE whereas this must be done manually in C-side.  Since the number of hypotheses scales linearly in the number of genes and quadratically in the number of cell types, the number of hypothesis tests can easily exceed 1 million limiting the power of traditional multiple testing approaches.  These differences lead niche-DE to detect more relevant genes than C-side, as shown on the liver metastasis example, even when the effective niche matrix computed by niche-DE is used as input to C-side.”

• The images in Fig. 4 and fig. 5 in the merged PDF file are low resolution and blurry, making it difficult to review the details. High-resolution H&E images for these tissue sections are not provided, and the pathologist annotations and color key are also missing.

We have added the annotations in the revision. High resolution images in supplementary figure 9.1.  Our results only hinge on features that are easily viewable in the images, such as tumor regions and fibrogenesis.

• For spatial transcriptomic data at single-cell resolution, such as the CosMx data, it is unclear how the authors handled doublets and gene dropout issues, which are essential for both niche-DE and niche-LR analysis. A more detailed explanation of the approach taken to address these challenges would be helpful for readers.

I am guessing that by doublets, the reviewer is referring to those caused by errors in cell segmentation.  Doublets containing two cells of the same type is not an issue since we normalize to account for differences in library size. The user can also detect doublets using algorithms such as Doublet Finder, Scrublet, Solo.  This should be done prior to application of nicheDE.  We didn’t find that to be necessary, as in the data sets we analyzed this didn’t seem to be an issue.

Perhaps the reviewer may also be referring to technologies such as SlideSeq V2, which are not single cell resolution.  For such data, we always apply deconvolution first, which would detect the presence of two cell types if they occupy the same grid region.  niche-DE operates on the deconvolution results, and does not assume that each square/spot is a single cell.

For gene dropout, this is why we used a negative binomial model, which has been well studied as a useful model for sequencing based single cell data, where dropout has been a pervasive issue.  We added references to existing literature that supports these models in the Results section of the revision.  Note that we are considering only genes that are in the top 25% of expressed genes, so dropout is less of an issue for these genes. Niche-DE looks at average effects across many cell type- cell type interactions so despite the dropout in a subset of the cells, there is still sensitivity for many niche-DE signals.

• Regarding the index and niche cell type annotations, what is the level of granularity? Does it focus on major cell types (e.g., fibroblasts, T cells, B cells, macrophages), or can it go a big further to distinguish specific cell states (e.g., cell states such as M2-like macrophages, regulatory T cells, exhausted T cells, naïve T cells, etc.)? Understanding cell-cell communications between various cell states is more important compared to just focusing on major cell types.

This is a good question.  The granularity of Niche-DE is determined by the granularity of cell types in the single cell reference that the initial cell type labeling/deconvolution (depending on whether the data is single cell or lower resolution) step can confidently separate.   This is an aspect where having single-cell resolution data is really advantageous:  The current deconvolution methods are still limited in their accuracy in teasing apart subtle cell types, and thus directly observing single cells may allow us to start a more fine-grained cell labeling.  As such, we added the following to the Results section under equation (2): “We stress that in the case of spot-level data, a good deconvolution must be performed. This is because errors in the deconvolution will lead to an incorrect effective niche calculation which is the primary covariate in the niche-DE model. Therefore, we assume that a satisfactory deconvolution has been performed prior to performing niche-DE.”

Also, niche-DE depends on there being an adequate “sample size” of spots containing a cell type. We address this in the discussion by adding the following text: “A central factor in the sensitivity of niche-DE for detecting niche genes between a given cell type pair is its rate of co-occurrence.  If a cell type is rare in the spatial sample, then niche-DE is unlikely to find any signal due to insufficient sample size.  Niche-DE will also be under-powered if two cell types never co-localize, or always co-localize.  We need to observe the two cell types both together and far apart, the latter providing the contrast for differential expression.  For spot-level data, a simple rule that we recommend is that there be at least 30 spots that contain the niche cell type and have the index cell type in its niche.”

That being said, if the initial deconvolution step can only distinguish broad cell type categories, then niche-DE can be used to further identify subtype-specific spatially-associated genes as exemplified in Fig.4G/H and Fig.5C where niche-DE finds two different types of tumor-associated fibroblasts and two different types of macrophages. As such, we expect that a common application of niche-DE would be to start with the resolution of cell types as estimated by the initial deconvolution/labeling, and then identify genes that serve as markers to further improve the cell type resolution by finding spatially-localized subtypes.

• Regarding niche differential expression (niche-DE) analysis, it is important to understand how the spatial niches are defined. What is the relationship between spatial niches and annotated cell types? Is a spatial niche an aggregate of a certain type of cells (with 100% cell type purity within a spatial niche)? or is it an aggregate of cells with mixed cell types? In the real-world scenarios, many immune cell types do not always cluster together and form a pure niche; instead, they are often intermixed. A clear explanation of the spatial niche definition would be helpful for readers.

Thanks for this question, and we agree that the definition needs to be more clear.  In the results section we add the text:  “In summary, we characterize the spatial niche of a cell as a T dimensional vector where index j measures how much of cell type j is in the neighborhood of the index cell. For example, a cell that is surrounded by tumor cells will have an effective niche that is 0 in all indices except for the one that corresponds to tumor cells. Meanwhile, a cell with a neighborhood dense in different immune cell types will have an effective niche with many non-zero entries. The exact density of each cell type within the neighborhood will determine the magnitude of the entry in the effective niche.”

• When integrating data from multiple tissue slides and across patients, how does niche-DE account for tissue- and slide-batch effects? It is important to assess the performance of niche-DE in correcting tissue- and slide-batch effects.

We agree that batch effects in spatial transcriptomics data still needs further study, and it’s not clear how well methods developed for single cell and bulk sequencing work in this setting. While it is unknown exactly how batch effects manifest in spatial transcriptomic data, we decided to address them by adding a batch indicator in our niche-DE regression for each gene. When there are multiple samples, there would be multiple batch indicators, one for each sample. If one believes that certain groups of observations will have different batch effects, they can add batch indicators for these groups as well.  This allows each gene to have a batch specific intercept term.   This is described in Methods, under the section “Joint analysis of multiple spatial transcriptomic data sets”.

• Compared to spatial domains defined based on spatially variable gene expression analysis, what are the main advantages of niche-differential expression analysis? the study does not provide a comparison with existing methods on spatial clustering analysis. clarifying the strengths of niche-DE over these methods would help emphasize its value.   Deciphering spatial domains (i.e., regions with similar spatial expression patterns) is one of the great challenges from STs.

Yes, we agree that identifying and characterizing spatial domains is one of the great challenges of ST data analysis.  From what we understand about spatial domains:  These are regions on a slide that have a specific cell type composition/configuration.  Spatially variable genes (SVG) can be used to identify spatial domains.  The goal of niche-DE, on the other hand, is to characterize how one cell type being in the vicinity of another cell type changes the expression of genes in that cell type.  So it is a relative analysis, looking at how the colocalization of two cell types *changes* their expression, conditioned on their cell type.  This is a different goal from SVG and spatial domain analysis.  We believe the two types of methods would complement each other.

Take, for example, the liver metastasis example shown in the paper.  An SVG analysis allows us to identify cancer-cell marker genes, which localize to the regions of the slide containing the malignancy.  The SVG analysis also allows us to detect fibroblast marker genes, which point us to the regions of desmoplasia which are enriched for fibroblasts.  However, if we wanted to know how fibroblasts change when they are near tumor cells, we would need to compare those fibroblasts whose niche is enriched for tumor cells to those that are further away from tumor cells.  In the data, fibroblasts are not very abundant and their spatial pattern is not as obvious:  Some fibroblasts are in the normal regions, some are in the tumor regions next to tumor cells, others are in desmoplastic regions surrounded by other fibroblasts and immune cells.  Niche-DE is able to quantify the specific effect of tumor cells on fibroblasts, accounting for the effects of other cell types in the niche.  Such an analysis is different and complementary to spatial domain detection.

In the revision, we highlight the difference between niche-DE, SVG and colocalization analysis in the Introduction and Discussion sections, as mentioned in the reply to the third bullet point above.

• The manuscript claims that their methods can also be applied to ROI-based spatial transcriptomics data, but it does not provide any data on ROI-based spatial transcriptomics data.

Sorry for this confusion, by ROI-based spatial transcriptomics data, we were mainly referring to spot- and other grid-based spatial transcriptomics data where each observation is a lower-resolution region on the slide rather than a single cell.  In the revision, we have deleted the phrase “ROI-based”.

• When applying niche-DE to lower resolution spot-level data, the authors deconvolved each sample using the single cell reference data set. If the spot-level ST data lack matched single-cell RNA-seq data, will this limit the application of niche-DE? Generating paired single-cell and spatial data for the same samples can be very challenging, due to high cost and insufficient tissue availability. In addition, most spatial data are generated using FFPE tissues, which cannot be used to generate single-cell RNA-sequencing data. If niche-DE can still be used to analyze the spot-level ST data without matched single-cell data, what deconvolution method can be employed? It would be helpful if the authors could benchmark their methods using existing datasets. additionally, how are unique gene signatures defined for deconvolution analysis? it is important to consider that some markers can be expressed by more than one cell types.

This is also a good point.  The mCRC Visium data that we analyzed (Figures 4, 5) does not have a matched single cell reference, and we had to resort to deconvolution using a public single cell atlas.  This shows that deconvolution of major cell types is feasible in this case.  We address the options when a matched scrna-seq reference is not provided in the discussion, with the added text:  “For spot-level spatial transcriptomic data, an important pre-processing step prior to performing niche-DE is deconvolution to estimate the cell type proportions within each spot. The accuracy by which niche-DE can detect cell-type specific niche-DE genes relies on the accuracy by which those cell types can be distinguished. Matched single cell data is not required for deconvolution as a single cell reference atlas of the same tissue can be used to perform deconvolution in most cases.  This was illustrated in our analysis of the liver metastasis data, for which there is no matched single cell RNA sequencing data.   “

Another option, when there is no matched single cell reference, is to rely on reference-free deconvolution methods such as ST-deconvolve.  When using a reference-free deconvolution technique, it is important to understand that conclusions will be made with regards to the cell type profiles found by the deconvolution. Therefore, it is important to match these profiles to known cell types post-hoc.

Deconvolution methods such as SpatialDWLS or RCTD use the marker gene sets for each cell type in deconvolution. Because most cell types will differ on the majority of their marker gene sets, deconvolution should not be affected by markers that are expressed by multiple cell types. For cell types that are more similar, such as T cell subtypes, deconvolution is more challenging.  We would caution applying niche-DE on deconvolution results that are low quality.   However, as we have shown with the analysis of tumor associated macrophages, starting with a deconvolution into coarse cell types, niche-DE can be used to identify marker genes for  cell subtypes provided they reside in different niches.  Our edits in the revision, on this point, are already highlighted in the replies to the previous bullet points.

• The Discussion is largely restating their results.

The discussion is now rewritten to include many aspects of niche-DE that are not in the results, such as the importance of the deconvolution step, the difference between niche differential expression and cell-cell colocalization analysis, the difference between niche differential expression and spatially variable gene analysis, and possible extensions of niche-DE.

Minor comments:

• Lines 23-24 and Line 32, the references cited are somewhat outdated, and recent significant publications have not been included.

This section has been updated to the latest references (citation 10-26 in the manuscript).

• For the CosMx SMI NSCLC data used in this study, how many samples and tissue sections are used?

In the Datasets and Data Analysis section we have added the details: We obtain Nanostring CosMx SMI non-small cell lung cancer data from [16]. We used Lung sample 9 which contains two tissue sections measuring 960 genes with 87684 and 139735 cells and 22 cell types. Each sample spans a 5mm by 4.25mm area.

Reviewer 2

Major comments:

* The analysis for spot-level data (e.g. 10x Genomics Visium) relies on first applying spot-level deconvolution to identify the cell types per spot. Could the authors provide some discussion of what would happen if the spot-level deconvolution does not perform well?

This is a good point, and we need to admit that niche-DE depends on a good quality spot deconvolution.  If the deconvolution is not good, niche-DE will not give good results. The deconvolution output determines what gene expression we expect to see at each spot.  For example, suppose a deconvolution method labels all fibroblasts as macrophages and vice-versa. Then all macrophage marker genes will be labeled as fibroblast niche genes since the “fibroblasts” will be expressing these genes much more than they are expected to.  As another example, if the deconvolution can not distinguish between subtypes of lymphocytes in a spot, then we wouldn’t be able to call lymphocyte subtype-specific niche-DE genes.

In the revision, we emphasized the importance of the initial deconvolution step at two places.  In the Results, we added the text: “We stress that in the case of spot-level data, a good deconvolution must be performed. This is because errors in the deconvolution will lead to an incorrect effective niche calculation which is the primary covariate in the niche-DE model. Therefore, we assume that a satisfactory deconvolution has been performed prior to performing niche-DE.”

In the discussion we add the text: “An important pre-processing step prior to performing niche-DE is deconvolution. Without a good deconvolution, niche-DE may not return coherent results. As an extreme example, suppose a deconvolution method labels all fibroblasts as macrophages and vice-versa. Then all macrophage marker genes will be labeled as fibroblast niche genes since the “fibroblasts” will be expressing these genes much more than they are expected to.”

* Related to the previous point, it would also be useful to include some more discussion of the types of data that the method is applicable to. For example, the cancer and cerebellum examples in the text are characterized by a diversity of major cell types. Would the method work in datasets from other biological systems that contain more subtle cell type differences (e.g. subpopulations of neurons in the cortex)?

Thanks for this good suggestion.  We should emphasize that in the liver metastasis example, there are subtle cell type differences (e.g. two different types of tumor-associated fibroblasts and two different types of macrophages) that are uniquely identified by niche-DE.  In this case, the initial deconvolution step only distinguished broad cell type categories, and niche-DE is used to further identify subtype-specific spatially-associated genes.  We expect that a common application of niche-DE would be to start with the resolution of cell types as estimated by the initial deconvolution/labeling, and then identify genes that serve as markers to further improve the cell type resolution by finding spatially-localized subtypes.

In some cases, one may want to distinguish subtle cell subtypes at the start, and characterize interactions between them.   Indeed, the sensitivity of niche-DE for cell-type specific genes depends on the accuracy with which those cell types can be deconvolved/labeled in the spatial data.  For lower resolution spot-based spatial data, this depends on whether these subtle cell types can be found by deconvolution.  For single-cell resolution data, this depends on the accuracy by which these cell types are labeled.  Sensitivity depends, of course, on the strength of the niche signal for a gene.  Thus, if there is high error in the deconvolution of a cell type, yet the niche effect on a gene is strong, it is still possible for niche-DE to detect it.

One related caveat is that if a cell type is rare in the spatial sample then niche-DE is unlikely to find any signal due to insufficient sample size. In order to find a signal between two cell types, we recommend that there be at least 30 spots that contain the niche cell type and have the index cell type in its niche.

In the revision, in addition to the edits highlighted in the response to the previous comment (“... the accuracy by which niche-DE can detect cell-type specific niche-DE genes relies on the accuracy by which those cell types can be distinguished …”), we also added:  “A central factor in the sensitivity of niche-DE for detecting niche genes between a given cell type pair is its rate of co-occurrence.  If a cell type is rare in the spatial sample, then niche-DE is unlikely to find any signal due to insufficient sample size.  Niche-DE will also be under-powered if two cell types never co-localize, or always co-localize.  We need to observe the two cell types both together and far apart, the latter providing the contrast for differential expression.  For spot-level data, a simple rule that we recommend is that there be at least 30 spots that contain the niche cell type and have the index cell type in its niche.”

* The runtime / computational scalability of the method are not clear. It would be useful for readers to include some results demonstrating the runtime / computational scalability, and providing some details on the largest dataset sizes that can feasibly be analyzed (e.g. number of cells and genes), and the type of computational hardware required (laptop or high-performance compute cluster, number of cores).

In the revision, we improved the computational scalability of niche-DE through parallelization, and we added detailed time tests.  Now, the niche-DE R package can parallelize over the number of cores available on your processor.  The time tests are shown in Figure 2D with the following text: “To benchmark runtime, we perform niche-DE across three different kernel bandwidths in parallel across 4 cores. We then vary the number of cell types, the number of observations, and the number of genes observed. We observe that the runtime increases linearly with the number of cells (spots) and the gene set size while it increases quadratically with the number of cell types (Fig.2 D) As a reference, it took 1 hour to analyze a dataset with 10,000 cells, 10,000 genes, and 8 cell types.”

Note that in practice, the runtime depends on how many pairs of cell types sufficiently collocalize since we filter out niche-DE interactions between rarely collocalizing cell types. It also depends on what percentage of genes we analyze for each index cell type. By default, niche-DE looks at the top 20% of expressed genes for each cell type.  In this sense our simulations represented upper bounds on the runtime since all cell types colocalized sufficiently and we analyzed the top 90% of genes for each cell type. Crashes on Rstudio began to occur when running niche-DE with 12 cell types, 50,000 spots, and 10,000 genes so we would recommend to either decrease the number of cores or to use a cluster for any datasets larger than this.

In the Methods section, we also added details on exactly how runtime was calculated by adding the text: “Under the generic model with no niche effects, we perform niche-DE across three different kernel bandwidths in parallel across 4 cores on a laptop. We varied the number of cell types between {4,8,12}, the number of observations between {1000,2000,3000,4000,5000,10000}, and the number of genes observed between {1000,5000,10000}. To do this, we first took a 10X Visium dataset of metCRC with 4 cell types. To generate new cell types, we replicated the existing cell types to create cell subtypes that were identical to the 4 original cell types. Thus, each cell type either had 0,1, or 2 replicates depending on the simulation. We then sampled either 1000, 5000, or 10000 randomly from the genome, and simulated a new Visium dataset by sampling new cells at random to populate spots. The location of these spots was generated by sampling the coordinates from the original Visium dataset with replacement. Afterwards, each cell’s expression would be drawn from a negative binomial with the mean gene expression equal to the average expression found by a single cell reference.”

* While the authors have set up an online vignette / tutorial to demonstrate usage of the method, the examples in this tutorial currently either return zero results (niche-DE genes and marker genes) or give errors (pathway enrichment and ligand-receptor analysis) when using the example dataset provided (raw data matrices). It would be useful to provide a tutorial that generates some meaningful results and/or plots, which users can then adapt for their own analyses.

Sorry for the errors in the vignette. Analyses are now updated to not give errors.

* Code and data to reproduce the analyses in the manuscript are unavailable. These should be provided in publicly accessible repositories.

We have added code and data to reproduce our analyses with Visium  in figures 4,5, and 6 at:

https://www.dropbox.com/sh/9mgdo8c4c42sd3x/AACFpQYVqvDszH0C_IBny8MNa?dl=0.

To make it as simple as possible, we only included the niche-DE objects and the corresponding analyses done using the niche-DE package. The raw data for the colorectal cancer metastasis and kidney fibrosis analyses are available from the data sources listed in their previous publications:

The raw data for liver metastasis Patients 4 and 5 are being processed for upload onto GEO.

 If you would like the processed data in any other form, please let us know.

Minor comments

* A short description / definition of the term "niche" in the introduction would be helpful.

Thanks for this suggestion.  In the introduction we add the text: “In our analyses, we define a cell’s niche as the cell type composition of its local neighborhood, where what is ``local’’ and what is not is determined by a kernel function. We expect that a cell’s niche affects its gene expression profile, and to this end, propose a new statistical procedure called niche-differential expression (niche-DE) analysis.”

* Installation and loading of the package in R returns a warning message, which should be addressed.

This warning has been addressed.

* Some citations are missing in the last paragraph of the introduction (currently showing placeholders "[CITE]").

All citations have been addressed.

**2^nd^ round**

**Reviewer 2**

The authors have addressed the main comments raised during the previous round of reviews, including new analyses and new text in the manuscript.

One comment was not fully addressed, regarding code and data availability. The previous review comments noted that code and data to reproduce the analyses in the manuscript were unavailable and should be provided in publicly accessible repositories. The authors partially addressed this by providing code and data via a Dropbox link. However, Dropbox links are unstable (e.g. they break if any files are updated or any new files added). These files should be moved to a persistent publicly accessible repository, e.g. GitHub for code and either GitHub or Figshare for data.
